# Art of the Kill: Designing and Testing Viral Inactivation Procedures for Highly Pathogenic Negative Sense RNA Viruses

**DOI:** 10.3390/pathogens12070952

**Published:** 2023-07-19

**Authors:** Judith Olejnik, Adam J. Hume, Stephen J. Ross, Whitney A. Scoon, Scott Seitz, Mitchell R. White, Ben Slutzky, Nadezhda E. Yun, Elke Mühlberger

**Affiliations:** 1 Department of Virology, Immunology and Microbiology, Chobanian & Avedisian School of Medicine, Boston University, Boston, MA 02118, USA; jolejnik@bu.edu (J.O.); hume@bu.edu (A.J.H.); sjross@bu.edu (S.J.R.); wmanhart@bu.edu (W.A.S.); scottallenseitz@gmail.com (S.S.); mitchw@bu.edu (M.R.W.); 2 National Emerging Infectious Diseases Laboratories, Boston University, Boston, MA 02218, USA; bslutzky@bu.edu (B.S.); yun.nadya@gmail.com (N.E.Y.); 3 Department of Biochemistry and Cell Biology, Chobanian & Avedisian School of Medicine, Boston University, Boston, MA 02118, USA

**Keywords:** virus inactivation, TRIzol, aldehydes, heat, Ebola virus, Nipah virus, Lassa virus, negative sense RNA viruses, Select Agents, BSL-4

## Abstract

The study of highly pathogenic viruses handled under BSL-4 conditions and classified as Select Agents frequently involves the transfer of inactivated materials to lower containment levels for downstream analyses. Adhering to Select Agent and BSL-4 safety regulations requires validation or verification of the inactivation procedures, which comes with an array of challenges for each method. This includes the use of cytotoxic reagents for chemical inactivation and defining the precise inactivation parameters for physical inactivation. Here, we provide a workflow for various inactivation methods using Ebola, Nipah, and Lassa viruses as our examples. We choose three distinct inactivation methods (TRIzol/TRIzol LS, aldehyde fixation using different fixatives, and heat) to highlight the challenges of each method and provide possible solutions. We show that, whereas published chemical inactivation methods are highly reliable, the parameters for heat inactivation must be clearly defined to ensure complete inactivation. In addition to the inactivation data, we also provide examples and templates for the documentation required for approval and use of inactivation SOPs, including an inactivation report, the procedure sections of developed SOPs, and an electronic inactivation certificate that accompanies inactivated samples. The provided information can be used as a roadmap for similar studies at high and maximum containment laboratories.

## 1. Introduction

Biosafety level 4 (BSL-4) laboratories provide the highest standard of biosafety and biosecurity to enable the safe handling of highly pathogenic infectious agents. This includes both protecting the environment and protecting the laboratory workers. Typically, BSL-4 agents are exotic zoonotic viruses that cause serious, often fatal, diseases in humans, are easily transmitted through aerosols or close contact with infected patients, and for which few to no treatment options are available. Viruses classified as BSL-4 include the entire filovirus family with Ebola virus (EBOV), Marburg virus, and Sudan virus; the arenaviruses with Lassa virus (LASV), Lujo virus, and the pathogenic New World arenaviruses; the nairoviruses with Crimean Congo hemorrhagic fever virus; and the henipaviruses with Hendra and Nipah virus (NiV) [1]. These viruses are also classified as Select Agents by the United States Federal Select Agent Program (FSAP) that is jointly comprised of the Centers for Disease Control and Prevention/Division of Select Agents and Toxins and the Animal and Plant Health Inspection Service/Division of Agricultural Select Agents and Toxins. Because these viruses cause deadly diseases in humans, it is of utmost importance to ensure the complete inactivation of any research material that may be removed from the BSL-4 space for further analysis at a lower biosafety level. FSAP regulations regarding the inactivation of materials containing Select Agents have been instituted to both protect the safety of laboratory workers handling such materials, as well as the public and the environment, from exposure to Select Agents.

Although there are multiple studies published that describe the chemical or physical inactivation of Select Agents and BSL-4 viruses, FSAP regulations still require that procedures used to demonstrate the chemical or physical inactivation of agents must be validated or verified in-house (at each research institution) to confirm their complete inactivation [2]. These regulations have been implemented because the complete inactivation of a given pathogen depends on numerous parameters, including physicochemical characteristics of the virus, viral load, cell number, volume, sample composition, exposure time, temperature, concentration of the inactivating chemical, limit of detection of the testing modality, etc.

In addition to defining the parameters underlying their respective inactivation procedure and validating the procedure itself in the laboratory, there is also an administrative part that includes writing the inactivation reports and Standard Operating Procedures (SOPs) for review by institutional, local, and federal regulatory agencies, such as biosafety boards, Institutional Biosafety Committees (IBC), and local or federal Health Commissions. Finally, after the inactivation procedure has been approved, Select Agent regulations require that each inactivated sample is accompanied by an inactivation certificate that contains specific details of the inactivation process to ensure that approved inactivation SOPs are followed.

Here, we provide example workflows for how to perform well-designed inactivation studies that are in compliance with FSAP biosafety regulations (7 CFR Part 331, 9 CFR Part 121, and 42 CFR Part 73) using EBOV, NiV, and LASV as our test viruses. Inactivation studies for these viruses involve substantial material costs and require significant amounts of research time spent in BSL-4 containment and should therefore be carefully designed. Before starting an inactivation study, it is of utmost importance to define the parameters that will be tested. This includes the type of tested material (e.g., animal tissue, adherent cells, suspension cells, or viral particles); the maximum amount of tested material (e.g., tissue weight, number of cells, or number of infectious viral particles); sample volume; sample composition (e.g., protein content); and the inactivation conditions. Testing the entirety of each sample (e.g., using all cells from a flask of cells fixed with formalin) provides confidence that even small amounts of remaining infectious virus, if present, will be detected. Conversely, testing only a portion of the sample, as may be necessary if testing highly diluted samples, leaves a risk of not detecting low amounts of infectious virus. In addition, the inclusion of key process controls, limit of detection analyses, and safety margins all serve to ensure that the validated inactivation procedure is robust. In this study, we highlight some of the challenges of chemical inactivation using toxic reagents (TRIzol and TRIzol LS) or fixatives (formalin, paraformaldehyde, and glutaraldehyde) and physical inactivation using heat with a focus on in vitro samples. We further provide examples of the documentation needed for inactivation approval that can be used as templates for performing inactivation procedures for other viruses or different procedures. We also include an example electronic inactivation certificate that serves to both meet FSAP regulations regarding record keeping of the inactivation of samples and for internal documentation purposes.

## 2. Materials and Methods

### 2.1. Biosafety Statement

All work with EBOV, NiV, and LASV was performed in the BSL-4 facility of Boston University’s National Emerging Infectious Diseases Laboratories (NEIDL) following approved SOPs in compliance with local (Boston Public Health Commission: Biological Laboratory Regulations) and federal (FSAP: 7 CFR Part 331, 9 CFR Part 121, and 42 CFR Part 73) regulations pertaining to handling BSL-4 pathogens and Select Agents.

### 2.2. Cell Lines

African green monkey kidney cells (Vero E6; ATCC Manassas, VA, USA; CRL-1586) were maintained in Dulbecco’s modified Eagle’s medium (DMEM; Gibco/Thermo Fisher Scientific, Waltham, MA, USA) supplemented with L-glutamine (200 mM; Thermo Fisher Scientific, Waltham, MA, USA) and 10% fetal bovine serum (FBS; R&D Systems, Minneapolis, MN, USA). Cell culture medium was supplemented with either penicillin (50 U/mL; Thermo Fisher Scientific, Waltham, MA, USA) and streptomycin (50 mg/mL; Thermo Fisher Scientific, Waltham, MA, USA) or 100 µg/mL Primocin (Invivogen, San Diego, CA, USA). Cells were grown at 37 °C and 5% CO_2_.

### 2.3. Virus Propagation

EBOV (isolate Mayinga, GenBank number NC_002549.1), NiV (strain Bangladesh, GenBank number NC_002728.1), and LASV (strain Josiah, GenBank numbers NC_004296.1 and NC_004297.1) were kindly provided by H. Feldmann, NIH NIAD Rocky Mountain Laboratories, Hamilton, MT, USA. Recombinant EBOV expressing green fluorescent protein from an additional transcription unit (EBOV-GFP) [3] was kindly provided by the World Reference Center for Emerging Viruses and Arboviruses (WRCEVA), UTMB, Galveston, TX, USA. EBOV expressing ZsGreen (GenBank number KR781609.1) was generated in our laboratory and was described before [4]. Recombinant vesicular stomatitis virus (VSV) expressing EBOV glycoprotein (GP) in place of VSV G and expressing GFP as an additional open reading frame (rVSV-GP_Z_-GFP) was described previously [5]. All viruses were propagated in Vero E6 cells in cell culture medium supplemented with 2% FBS. Purified stocks were generated using ultracentrifugation through a 20% sucrose cushion as previously described [6]. Virus titers were determined in Vero E6 cells by a tissue culture infectious dose 50 (TCID_50_) assay using the Spearman and Kärber algorithm [4,7].

### 2.4. TRIzol and TRIzol LS Inactivation Testing

To determine if diluting TRIzol LS (Thermo Fisher Scientific, Waltham, MA, USA) eliminates cytotoxicity, TRIzol LS was diluted in media (DMEM supplemented with 2% FBS, L-glutamine, and 100 µg/mL Primocin) at a ratio of 1:67 (45 µL TRIzol LS added to 3 mL media). Cells were treated either with media or diluted TRIzol LS and then mock-infected or infected with rVSV-GP_Z_-GFP at an MOI of 0.1. Samples were monitored for a cytopathic effect (CPE) and GFP expression at 2 days post-infection (dpi).

To test the ability of TRIzol (Thermo Fisher Scientific, Waltham, MA, USA) to inactivate EBOV-, NiV-, or LASV-infected cells, Vero E6 cells seeded in T175 flasks were mock-infected or infected with the respective virus (see Table S1 for virus-specific conditions). The progress of the infection was monitored by analyzing fluorescence and/or CPE. At the indicated days post-infection (Figure S1), when complete infection of cells or pronounced CPE was observed, cell supernatants were removed, and the cells were scraped into 20 mL of PBS (phosphate-buffered saline; Thermo Fisher Scientific, Waltham, MA, USA), transferred into tubes, pelleted by low-speed centrifugation, and resuspended in 1 mL PBS or DMEM or TRIzol. To determine the cell number of the T175 flasks at the time of inactivation, an extra flask with cells was incubated for the same time and used to count the cells. The PBS/DMEM and TRIzol samples were vortexed, incubated for 10 min at room temperature, and purified using size exclusion columns (Amicon Ultra-0.5 Centrifugal Filter Unit 10 kDa; Millipore-Sigma, Burlington, MA, USA), as previously described [8]. The samples were eluted in 0.5 mL of PBS. The entirety of the used column eluates was used to infect 2 × 10^7^ Vero E6 cells seeded in T175 flasks. For the challenge samples, the respective virus was mixed with the column-purified eluate from TRIzol-treated non-infected cells and used to infect cells (see Table S1 for virus-specific conditions). Flasks were incubated for the indicated times (Figure S1) and checked for signs of fluorescence and/or CPE every 2–3 days. Cell supernatants were clarified by low-speed centrifugation, and the entire supernatant was used to infect Vero E6 cells seeded in T175 flasks. The flasks were checked for CPE every 2–3 days. At the indicated days post-infection (Figure S1), cell supernatants were clarified by low-speed centrifugation, and 0.2 mL was used to infect Vero E6 cells seeded in a 96-well plate. Three days post-infection, the cells were fixed with 10% formalin, and immunofluorescence analysis using virus-specific antibodies was performed to determine infection rates.

To examine the capability of TRIzol LS to inactivate EBOV, NiV, or LASV particles, 250 µL of mock media (PBS + 10% FBS or 250 µL DMEM + 2% FBS) or concentrated virus stock (see Table S1 for virus-specific conditions) in PBS + 10% FBS or 250 µL DMEM + 2% FBS were mixed with 750 µL of TRIzol LS. The ratio of TRIzol LS to the sample was 3:1 (*v*/*v*), as recommended by the manufacturer. The samples were vortexed, incubated for 10 min at room temperature, and purified using size exclusion columns (Amicon Ultra-0.5 Centrifugal Filter Unit 10 kDa) as previously described [8]. The samples were eluted in 0.5 mL of PBS. The entirety of the used column eluates was used to infect 2 × 10^7^ Vero E6 cells seeded in T175 flasks. For the challenge samples, the respective virus was mixed with the column-purified eluate from TRIzol LS-treated mock media (PBS + 10% FBS or 250 µL DMEM + 2% FBS) and used to infect cells (see Table S1 for virus-specific conditions). Passaging to a second T175 flask, the monitoring of cells, infection of a 96-well plate, and immunofluorescence analysis were performed as described for TRIzol testing (see also Figure S1).

### 2.5. Aldehyde Inactivation Testing

To evaluate the potential of formalin and paraformaldehyde (PFA) to inactivate EBOV-, NiV-, or LASV-infected cells, Vero E6 cells seeded in T175 flasks were mock-infected or infected with the respective virus (see Table S1 for virus-specific conditions). To test glutaraldehyde inactivation of EBOV-infected cells, Vero E6 cells seeded in T75 flasks were mock-infected or infected with EBOV-ZsGreen. The progress of the infection was monitored by analyzing fluorescence and/or CPE. At the indicated days post-infection (Table S1 and Figure S2), when complete infection of cells or pronounced CPE was observed, cell supernatants were removed and replaced with 35 mL (200 µL per cm^2^ of cell monolayer) of PBS, DMEM, 10% neutral buffered formalin (LabChem, Zelienople, PA, USA), 4% PFA, or 15 mL of 2% glutaraldehyde (200 µL per cm^2^ of the cell monolayer). Flasks were rotated to make sure the fixatives came into contact with all interior surfaces. Neutral buffered formalin (10%) was used within the expiration date. Glutaraldehyde (10%, Tousimis, Rockville, MD, USA) was diluted to 2% in PBS, stored at 4 °C, and used within the expiration date. In addition, PFA (32%, Electron Microscopy Sciences, Hatfield, PA, USA) was diluted to 4% in either DMEM without phenol red (Lonza Bioscience, Morrisville, NC, USA) or PBS, stored at 4 °C, and used within 6 months. Samples were incubated for 30 min, 1 h, or 6 h at 4 °C. After incubation, PBS, DMEM, or fixative was removed, and cells were rigorously washed four times with 20 mL of PBS to eliminate any cytotoxic effects of the used fixatives. Cells were scraped into 20 mL (T175) or 10 mL (T75) of PBS, transferred into tubes, pelleted by low-speed centrifugation, and resuspended in 1 mL PBS or DMEM. To determine the cell number of the flasks at the time of inactivation, an extra flask with cells was incubated for the same time and used to count the cells. The cells incubated in PBS, DMEM, 10% formalin, 4% PFA, or 2% glutaraldehyde were then used to infect Vero E6 cells seeded in T175 or T75 flasks the day prior. For the challenge samples, the respective viruses were mixed with formalin-, glutaraldehyde-, or PFA-treated non-infected cells and used to infect cells (see Table S1 for virus-specific conditions). The challenge samples were included to show that the respective virus can replicate in cells that were exposed to formalin-, glutaraldehyde-, or PFA-treated cells. Passaging to a second T175 or T75 flask, the monitoring of cells, infection of a 96-well plate, and virus-specific immunofluorescence analysis were performed as described for TRIzol testing (see also Figure S2).

In addition, the ability of 10% formalin and 4% PFA to inactivate EBOV in cell pellets was evaluated. Vero E6 cells seeded in T75 flasks were mock-infected or infected with EBOV-GFP (Table S1 and Figure S2) and processed at indicated times as described in the section above. Cells were detached using trypsin and pelleted by low-speed centrifugation. After removing the cell culture medium, the cell pellets were overlaid with 2 mL of 10% formalin or 4% PFA for fixation. Two milliliters of DMEM were used for the untreated control samples. After incubation for 6 h at 4 °C, cell pellets were washed 4 times with 2 mL PBS to remove any cytotoxic effects of the fixative. Cell pellets were resuspended in 1 mL DMEM and used to infect Vero E6 cells seeded in T75 flasks, as described in the section above, including an EBOV-GFP challenge sample (see Table S1 for virus-specific conditions). Passaging to a second T75 flask, the monitoring of cells, infection of a 96-well plate, and immunofluorescence analysis were performed as described for TRIzol testing.

To examine formalin inactivation of EBOV particles, 100 µL of concentrated EBOV-ZsGreen stock (see Table S1 for the conditions) in PBS or 100 µL of PBS were mixed with 100 µL of 20% formalin (Richard-Allan Scientific) for a final formalin concentration of 10% (*v/v*). The samples were incubated for 1 or 6 h at 4 °C and purified using Amicon size exclusion columns as previously described [8]. The samples were eluted in 0.5 mL of PBS. The entirety of the used column eluates was used to infect 2 × 10^7^ Vero E6 cells seeded in T75 flasks. Passaging to a second T75 flask, the monitoring of cells, infection of a 96-well plate, and immunofluorescence analysis were performed as described for TRIzol testing (see also Figure S2).

### 2.6. Heat Inactivation Testing

Prior to the heat inactivation study, we verified that the used heat block reached the required temperature using a Monacor DTM-506RS digital thermometer (Monacor International, Bremen, Germany) equipped with a wired temperature sensor. We excluded the Eppendorf Thermomixer from the study, because in our hands, the required temperature of ≥95 °C was not reached in the samples when the thermomixer’s maximal temperature was set to 99 °C. We therefore used the Advanced Dry Block Heater (VWR, Radnor, PA, USA) for our studies. To determine the temperature profile in the tubes, the temperature sensor was inserted through a hole in the lid of a 2 mL screw cap tube filled with 1 mL of water, and the tube was placed in a preheated heat block set to 120 °C. The temperature was measured for seven minutes. The tube was placed at different positions within the heat block.

To develop a heat inactivation protocol for EBOV-infected cells, 1 × 10^7^ Vero E6 cells seeded in T175 flasks were mock-infected or infected with EBOV (see Table S1 for virus-specific conditions). The progress of the infection was monitored by analyzing CPE. At 3 days post-infection (Figure S3), when pronounced CPE was observed, cell supernatants were removed, and the cells were scraped into 20 mL of PBS, transferred into tubes, pelleted by low-speed centrifugation, resuspended in 1 mL PBS, and placed in 2 mL screw cap tubes. Samples were incubated at room temperature or in the Advanced Dry Block Heater (VWR) set to 120 °C for 10 min. The temperature was verified by an internal heat block thermometer and an external thermometer placed in a water-filled tube in the heat block. Samples were not placed into the heat block until the external thermometer reached at least 99 °C. The heat-treated samples were then used to infect 2 × 10^7^ Vero E6 cells seeded in a T175 flask. For the challenge samples, EBOV was mixed with non-infected cells that had been incubated at 120 °C for 10 min, and the mixture was used to infect cells (see Table S1 for virus-specific conditions). The EBOV challenge sample was included to show that EBOV could replicate in cells that were exposed to heat-treated cells.

To determine the infection rate at the time of inactivation, Vero E6 cells seeded in chamber slides were infected in parallel with EBOV at the same MOI (MOI = 10). These infection control slides were fixed 3 days post-infection, at the time of virus inactivation, and analyzed for the presence of EBOV by immunofluorescence analysis. To determine the cell number of the T175 flasks at the time of inactivation, an extra flask with cells was incubated for the same time and used to count the cells (2.4 × 10^7^ cells after 3 days). Passaging to a second T175 flask, the monitoring of cells and infection of cells seeded in a 96-well plate, followed by immunofluorescence analysis, were performed as described for TRIzol testing (see also Figure S3).

To establish a heat inactivation protocol for EBOV-containing solutions (see Table S1 for conditions tested), 2.25 × 10^8^ TCID_50_ units of EBOV supplemented with FBS at a final concentration of 10% in a total volume of 1 mL PBS were incubated in the Advanced Dry Block Heater (VWR) set to 120 °C for 10 min. The temperature was verified as was done with the EBOV-infected cell samples. The samples were then used to infect 2 × 10^7^ Vero E6 cells seeded in a T175 flask. Passaging to a second T175 flask, the monitoring cells and infecting cells seeded in a 96-well plate, followed by immunofluorescence analysis, were performed as described for TRIzol testing (see also Figure S3).

### 2.7. Limit of Detection Analysis

To assess the sensitivity of our experimental design, limit of detection (LOD) analysis was performed. For these assays, 1, 10, or 100 TCID_50_ units of virus diluted in cell culture medium supplemented with 2% FBS were added to a T75 or T175 flask of Vero E6 cells. LOD samples were monitored for the presence of CPE and fluorescence (when analyzing EBOV-GFP or EBOV-ZsG) in the initial flasks (see Figure S4A for an overview of LOD sample processing). The entirety of the supernatants from the initial flasks were transferred to a second flask of Vero E6 cells. The cells were assessed for CPE and/or fluorescence, and supernatants were then transferred to 96-well plates of Vero E6 cells (Figure S4A). After 2–3 days of infection, 96-well plates were fixed with 10% formalin, and virus-specific immunofluorescence analysis was performed.

### 2.8. Immunofluorescence Analysis

Vero E6 cells seeded in 96-well plates (2 × 10^4^ cells/well) were infected with 200 µL of cell supernatant (3–4 wells per test sample), and three 1:10 serial dilutions were performed per well. Infected cells were incubated for the indicated times at 37 °C (Figures S1–S4) and fixed with 10% formalin. The fixed cells were permeabilized with 0.1% Triton X100 (Boston Bioproducts, Milford, MA, USA) for 5–10 min at room temperature; incubated in 0.1 M glycine (Boston Bioproducts, Milford, MA, USA) for 5 min at room temperature; and subsequently incubated in blocking buffer (2% bovine serum albumin, 0.2% Tween 20, 3% glycerol, and 0.05% sodium azide in PBS) or 5% goat serum (Jackson ImmunoResearch, West Grove, PA, USA) for 20–60 min at room temperature. After each step, the cells were washed three times in PBS. For the EBOV samples, cells were incubated overnight at 4 °C with a custom goat polyclonal antiserum directed against the EBOV VP35 protein (Antagene; 1:200 dilution in blocking buffer or 5% goat serum). For the NiV samples, cells were incubated overnight at 4 °C with polyclonal anti-NiV hyperimmune mouse ascitic fluid (BEI; 1:200 dilution in 5% goat serum). For the LASV samples, cells were incubated overnight at 4 °C with polyclonal anti-LASV hyperimmune mouse ascitic fluid (BEI; 1:200 dilution in 5% goat serum). The cells were washed four times in PBS and incubated with secondary antibodies plus 4′,6-diamidino-2-phenylindole (DAPI; Sigma-Aldrich at 200 ng/mL for nuclei staining) for 1 h at room temperature. EBOV samples were incubated with donkey anti-goat antibody conjugated with either AlexaFluor488 or AlexaFluor594 (Invitrogen; 1:200 dilution in blocking buffer or 5% goat serum). NiV and LASV samples were incubated with chicken anti-mouse antibody conjugated with AlexaFluor488 (Invitrogen; 1:200 dilution in 5% goat serum). Images were acquired using a Nikon TS100 Eclipse microscope and Nikon DS Qi1Mc camera with NIS Elements F software or with a Nikon Eclipse Ti2 microscope with a Photometrics Prime BSI camera and NIS Elements AR software.

### 2.9. Amicon Column Testing

To determine the virus loss during column purification, 0.5 mL NiV stock containing 2.5 × 10^8^ TCID_50_ units or 0.5 mL LASV stock containing 2 × 10^8^ TCID_50_ units was added to the size exclusion columns (Amicon Ultra-0.5 Centrifugal Filter Unit 10 kDa). Column purification was performed according to the manufacturer’s instructions. Columns were washed three times with PBS. The column content was resuspended in 0.5 mL DMEM supplemented with L-glutamine (200 mM), penicillin (50 U/mL), streptomycin (50 mg/mL), and 2% FBS and eluted from the columns. Titers of the virus suspensions eluted from the columns were determined by TCID_50_ assay in comparison to non-purified viral stocks (triplicate samples each).

### 2.10. Mycoplasma DNA PCR on BSL-4 Samples

To test BSL-4 cell supernatants or viral stocks for mycoplasma contamination, we used the QuickExtract™ DNA Extraction Solution (Epicentre Biotechnologies, Madison, WI, USA) in combination with heat inactivation to extract mycoplasma DNA for PCR analysis from the samples. QuickExtract™ DNA Extraction Solution is used to extract genomic DNA from diverse samples for PCR amplification. Cell lysis and DNA extraction include a 6-min incubation at 65 °C followed by a 2-min incubation at 98 °C. We adapted this protocol to our needs by replacing the second heat step with a 10-min incubation at 120 °C in the Advanced Dry Block Heater (VWR), which we use for heat inactivation. Briefly, 50 µL of cell supernatant or virus stock solution were mixed with 200 µL QuickExtract™ DNA Extraction Solution in a sealed screw top tube and incubated for 6 min at 65 °C, followed by 10 min at 120 °C. The Mycoplasma Detection kit from SouthernBiotech (Birmingham, AL, USA) was used according to the manufacturer’s instructions. *M. orale* DNA is included in the kit as a positive control and leads to a 503 bp PCR fragment. Mycoplasma-infected samples will generate PCR products between 448 and 611 bp, depending on the mycoplasma species that is present. An internal negative control (270 bp) is included to prevent false negatives due to PCR inhibitors.

## 3. Results

### 3.1. Setting up a Well-Designed Inactivation Study

**Figure 1:**
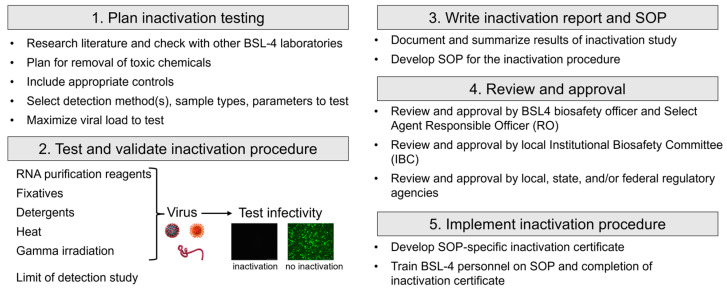
Workflow for inactivation testing.

Inactivation studies for BSL-4 pathogens and Select Agents are subject to a rigorous review process by local and potentially federal biosafety officers and regulatory agencies. In addition, depending on federal regulations that vary among countries, there might be a requirement to document and record the inactivation procedure for each inactivated sample that will be removed from the maximum containment laboratory. Complying with these regulations requires a well-designed workflow that covers the distinct steps from designing the inactivation study and developing a procedure-specific SOP to communicating the results to regulatory agencies and, upon approval, maintaining the necessary documentation (Figure 1). Each inactivation study comes with its own challenges. Chemical inactivation often requires the removal of cytotoxic reagents that interfere with testing the viability of inactivated viruses in cell culture [8,9,10,11,12,13]. Physical inactivation, such as gamma irradiation and heat, relies on highly specific parameters, including the sample volume, number of cells or viral particles, protein content, temperature, and inactivation time [5,14,15,16].

**Figure 2:**
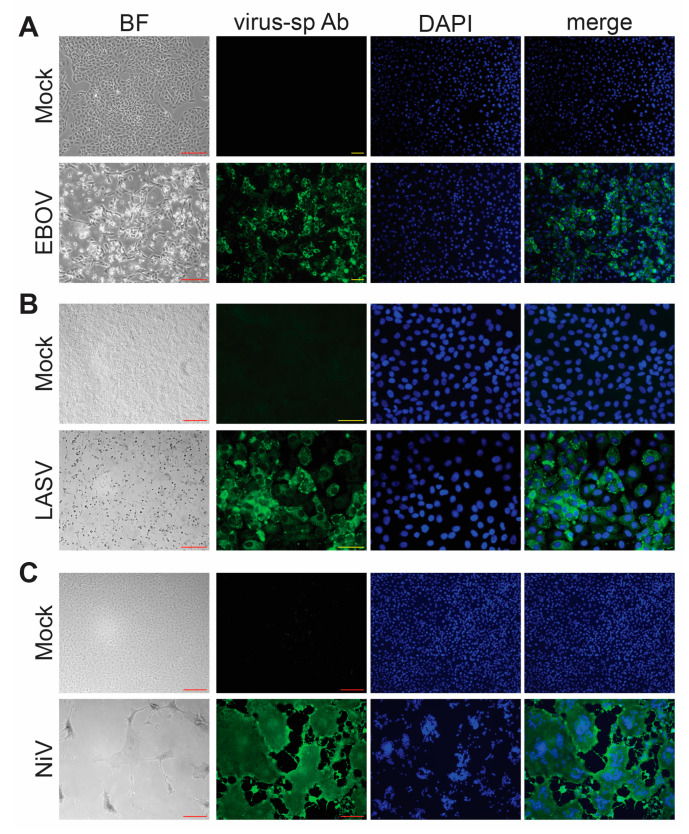
Virus detection. Brightfield (BF) microscopy and immunofluorescence analysis (IFA) of Vero E6 cells infected with (**A**) EBOV, (**B**) LASV, or (**C**) NiV. The infected cells show clear CPE under BF microscopy. Green, virus-specific antibody (virus-sp Ab) staining; blue, cell nuclei stained with DAPI. Red scale bars = 200 µm; yellow scale bars = 50 µm.

**Figure 3:**
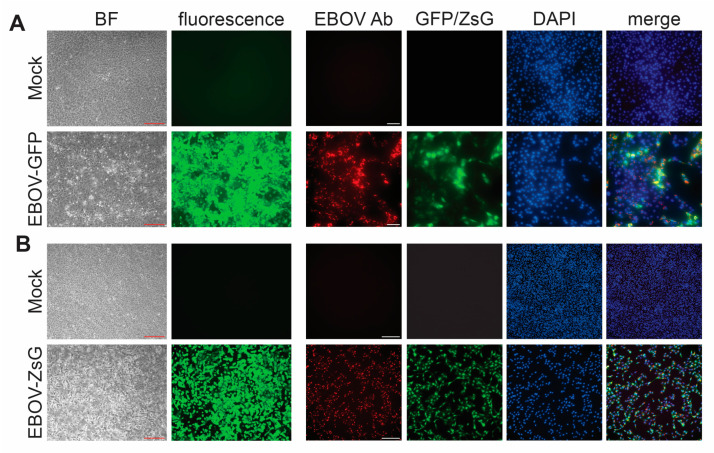
Detection of recombinant viruses expressing fluorescent proteins. Brightfield (BF) microscopy to assess the presence of CPE, fluorescence microscopy (green), and virus-specific immunofluorescence analysis (red) of Vero E6 cells infected with (**A**) recombinant EBOV expressing GFP or (**B**) recombinant EBOV expressing ZsGreen (ZsG). Cell nuclei were stained with DAPI (blue). Ab, antibody. Red scale bars = 200 µm; white scale bars = 250 µM.

Another important consideration when performing inactivation testing is the choice of which virus to use. FSAP regulations require separate testing for each virus family and each inactivation method [2]. For testing with wildtype viruses, cells are typically monitored for CPE (see Figure 2 for examples of CPE from EBOV, NiV, and LASV) as evidence of viral infection. Our inactivation studies were performed using Vero E6 cells, because these cells can be maintained in culture for several weeks without changes to their morphology, which makes them well suited for inactivation studies that rely on the development of CPE due to infection. For both EBOV and LASV infection of Vero E6 cells, we observed CPE, including cell rounding, cell detachment, increased light refraction of infected cells, and an overall reduction in cell density (Figure 2A,B). In comparison, NiV infection resulted in the formation of syncytia, which increased in size over time, and, eventually, cell detachment and reduced cell density (Figure 2C). This was accompanied by immunofluorescence analysis using virus-specific antibodies to ensure that the observed CPE is due to infection. In addition, immunofluorescence analysis enables the detection of viruses that cause mild or no CPE in infected cells. Disadvantages of immunofluorescence analysis include that it can only be used for end point analysis because the cells must be fixed and that it is not suitable for large cell numbers. These disadvantages can be overcome using recombinant viruses expressing a fluorescent reporter. Monitoring for fluorescence is an attractive approach for assessing the presence of a virus within a test sample (Figure 3).

A well-designed inactivation study includes several key controls. It is essential to include a limit of detection (LOD) analysis to demonstrate the sensitivity of the virus detection assays (see Section 3.2). Another important control is the inclusion of virus challenge samples. These challenge samples consist of non-infected cells that are treated in the same way as the inactivated, infected samples and are spiked with infectious virus after the inactivation procedure, including potential wash steps or the removal of toxic reagents, has been completed. These controls are critical for demonstrating that the procedure used for the removal of toxic reagents has no effect on the ability of subsequent cells to be infected after treatment.

In the sections below, we will provide examples of different inactivation procedures for various BSL-4 viruses, with a focus on potential challenges connected to the specific inactivation procedure. We have developed an inactivation testing rubric that is adaptable to different parameters and viruses tested. The overall scheme of each inactivation testing described in this report can be found in Figures S1–S3.

### 3.2. Limit of Detection (LOD) Analysis

To ensure that our testing protocol was sensitive and capable of detecting small amounts of infectious viruses, we performed LOD analyses alongside our inactivation testing. For this, low amounts of virus (1, 10, or 100 TCID_50_ units) were added to T175 flasks and processed exactly the same as the test samples (Figure S4A). The samples were monitored for CPE and analyzed using virus-specific immunofluorescence analysis. Recombinant viruses were additionally monitored for fluorescence. An example of LASV LOD analysis is included in Figure S4B. Notice the more abundant CPE with the 10 versus the 1 TCID_50_ unit LOD samples after 4 days with the example data (Figure S4B, first column). LOD analysis was performed for each virus and inactivation procedure in parallel to performing the inactivation experiments to ensure detection. We demonstrate the reliable detection of all used viruses at very low amounts (Table 1). In addition, these analyses ensure that a lack of virus detection during the experimental testing is due to the successful inactivation of the virus below the LOD.

### 3.3. Chemical Inactivation with Compounds for Nucleic acid Extraction: TRIzol and TRIzol LS

The inactivation of virus-containing cells, tissues, cell supernatants, or body fluids for the purpose of nucleic acid extraction and downstream analyses is often performed using guanidinium thiocyanate-containing buffers such as TRIzol and TRIzol LS (mixtures of phenol CAS # 108-95-2, guanidine isothiocyanate CAS # 593-84-0, and ammonium thiocyanate CAS # 1762-95-4) in slightly different ratios); MagNA Pure lysis buffer (guanidinium thiocyanate and Triton X-100 CAS #9036-19-5, Roche) [17]; or AVL or RLT (guanidinium thiocyanate, Qiagen). Note that prior inactivation testing has shown that AVL and RLT buffers require the addition of ethanol for complete viral inactivation [10,18]. We chose to test TRIzol (for infected cells) and TRIzol LS (for supernatants/virus stocks), because these buffers have been safely used for decades to inactivate virus-containing samples, do not require the addition of ethanol to completely inactivate samples containing virus, and can be used to stably store samples for long periods of time for later RNA extraction. Due to the strong cytotoxicity of these buffers, analyzing the potential infectivity of treated samples is challenging. One method to resolve this issue is to dialyze the samples, which removes small toxic compounds but retains any potential infectious virus [10]. Tangential flow filtration and diafiltration are newer methods available that take less space than traditional dialysis, although these approaches typically require equipment that not all laboratories may have available. A different approach to resolve the issue of TRIzol toxicity is by diluting TRIzol- and TRIzol LS-containing samples in cell culture media [19,20]. Using the same dilution factor (1:67) as described in [19], we observed that, although cells treated with diluted TRIzol LS did not show gross CPE, these cells were not capable of being infected with recombinant VSV-expressing EBOV GP (Figure S5). In contrast, Alfson and colleagues were able to infect the cells that had been exposed to diluted TRIzol LS with EBOV [19]. Importantly, this illustrates the need for including virus challenge samples, since cells exposed to diluted toxic reagents, though looking healthy, may not be permissive to viral infection anymore, which can lead to false-negative results in the inactivation study.

Another method for removing toxic chemicals while retaining any potentially infectious virus is to use size exclusion columns compatible with these buffers [20], which we outline here. We have used size exclusion column purification to remove TRIzol, TRIzol LS, or TCL buffer from EBOV-, NiV-, or LASV-infected cells or supernatants [8] (Table 2). As an example of these studies, we present here the data obtained for using TRIzol to inactivate NiV. Briefly, cells were infected with NiV at a high MOI and then harvested in TRIzol 2 dpi. To ensure that downstream-approved inactivation protocols would cover the inactivation of large numbers of cells and obviate the potential need for additional testing, we used T175 flasks of NiV- or mock-infected Vero E6 cells. Cells were scraped and resuspended in either 1 mL DMEM or TRIzol and incubated for 10 min at room temperature. Samples lysed in TRIzol were column-purified to eliminate the toxicity. We have previously shown that filtering EBOV-containing samples over size exclusion columns has minimal impact on virus titers [8] and now present similar data for both NiV and LASV (Figure S6).

**Figure 4:**
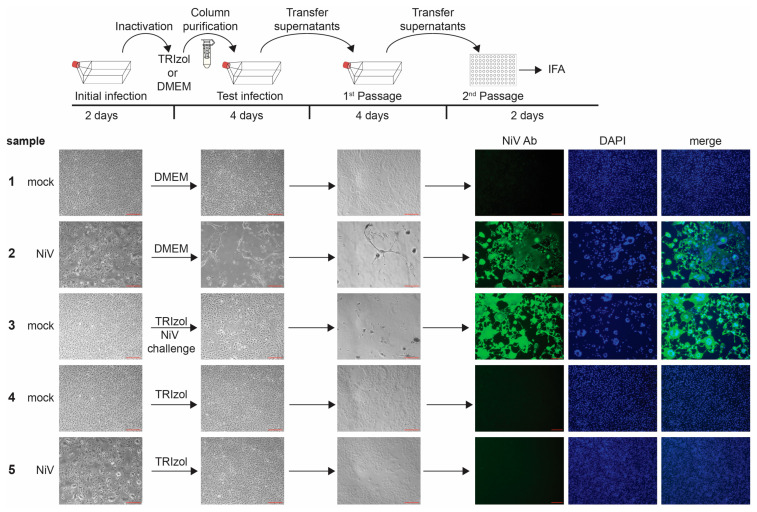
Inactivation of NiV with TRIzol. Top, schematic of the assay. Vero E6 cells seeded in T175 flasks were mock-infected or infected with NiV at an MOI of 0.5. At 2 days post-infection (dpi), brightfield images were taken to assess the presence of CPE in samples as a marker for viral infection (initial infection). Cells were harvested in either DMEM or TRIzol, column-purified, and transferred onto Vero E6 cells seeded in T175 flasks. Challenge samples were infected with NiV at MOI 0.01. Samples were monitored for CPE at 4 dpi (Test infection). Clarified supernatants were passaged onto Vero E6 cells seeded in T175 flasks. Cells were incubated for an additional 4 days and monitored for viral infection (1st Passage). Clarified supernatants were then used to infect Vero E6 cells seeded in 96-well plates and fixed at 2 dpi. IFA was performed using an anti-NiV antibody (green, 2nd Passage). Cell nuclei were stained with DAPI (blue). Scale bars = 200 µm.

Since cells were monitored for CPE (see Figure 2 for examples of CPE from wt EBOV, NiV, and LASV) as evidence of viral infection, we included a negative control consisting of mock-infected cells resuspended in DMEM and a positive control consisting of NiV-infected cells resuspended in DMEM (Figure 4, samples 1 and 2). To ensure that the columns removed the toxic components of TRIzol, mock-infected cells that were lysed in TRIzol and then column purified were included (Figure 4, sample 4). We also included a NiV challenge sample, in which mock-infected cells were lysed in TRIzol, processed over the columns, and added to cells along with infectious NiV (Figure 4, sample 3). Finally, NiV-infected cells were treated with TRIzol and column-purified to assess the ability of TRIzol to inactivate the virus. Processed samples were added to T175 flasks to minimize any effect of residual toxicity. This also facilitated the testing of 100% of each sample in compliance with FSAP regulations [2]. Cells were regularly monitored for CPE with images taken. Four days post-infection, the entirety of the supernatants from these flasks were transferred to fresh T175 flasks to facilitate the expansion of small amounts of virus, if present. Cells were again monitored for CPE and imaged at 4 dpi. A portion of these supernatants was then used to infect cells seeded in 96-well plates in order to perform virus-specific immunofluorescence analysis to confirm the CPE-based results. All samples showed the expected results, with only the positive control (NiV + DMEM) and NiV challenge samples showing CPE and staining for NiV (Figure 4, samples 2 and 3). The NiV-infected cells lysed in TRIzol showed complete inactivation (Figure 4, sample 5), consistent with previous results [12].

We used a slightly modified protocol for testing the ability of TRIzol LS to inactivate NiV. Concentrated virus was incubated with TRIzol LS, column-purified, and added to cells. Subsequent analysis was performed similar to the analysis of the TRIzol samples (Figure S1) and showed that TRIzol LS was able to inactivate NiV virions (Table 2), in line with previous results [12]. Similar analyses were also performed for EBOV- and LASV-infected cells and virus stocks, which showed complete inactivation in agreement with the published results (Table 2) [10,19,21,22].

In conclusion, our studies confirm that TRIzol and TRIzol LS reliably inactivate negative sense RNA viruses, including filoviruses, arenaviruses, and henipaviruses. We did not observe any differences in the ability of these chemicals to efficiently inactivate EBOV, LASV, or NiV. We also show that column filter purification is an easy and reliable method to remove toxic substances from viral samples treated with chemicals commonly used to inactivate viruses. A comprehensive analysis of different types of columns to remove distinct chemicals from SARS-CoV-2 samples and determine viral recovery rates was performed by Welch and colleagues [11], and we recommend consulting this report for selecting the most suitable column filtration system for a planned chemical inactivation study. Based on the inactivation results, we developed an agent- and procedure-specific SOP. The procedure section of this SOP has been added as the Supplementary Materials S1.

### 3.4. Aldehyde Inactivation

**Figure 5:**
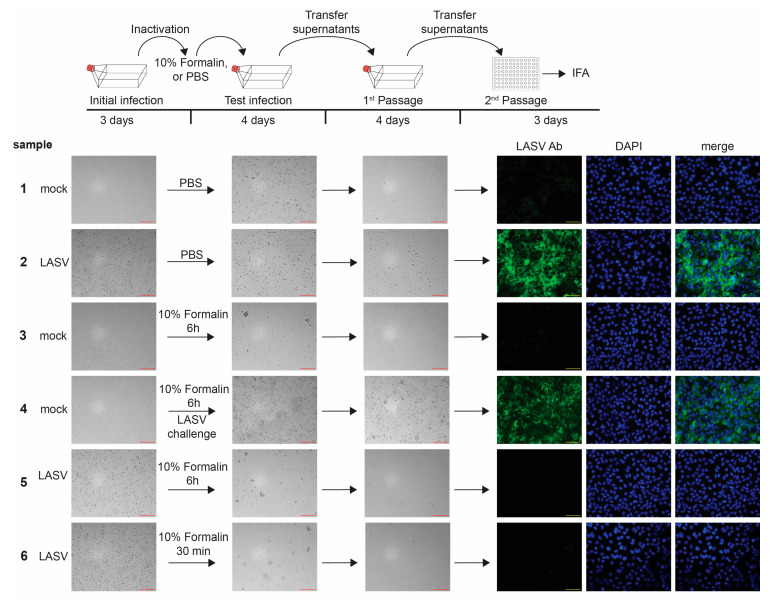
Inactivation of LASV with 10% formalin. Top, schematic of the assay. Vero E6 cells seeded in T175 flasks were mock-infected or infected with LASV at an MOI of 5. At 3 dpi, brightfield images were taken to assess the presence of CPE in samples as a marker for viral infection (Initial infection). Cells were fixed in 10% formalin or incubated in PBS, scraped, washed with PBS, and transferred onto Vero E6 cells seeded in T175 flasks. Challenge samples were infected with LASV at MOI 0.1. Samples were monitored for CPE at 4 dpi (Test infection). Clarified supernatants were passaged onto Vero E6 cells seeded in T175 flasks. Cells were incubated for an additional 4 days and monitored for viral infection (1st Passage). Clarified supernatants were then used to infect Vero E6 cells seeded in 96-well plates and fixed at 2 dpi. IFA was performed using an anti-LASV antibody (green; 2nd Passage). Cell nuclei were stained with DAPI (blue). Red scale bars = 200 µm; yellow scale bars = 50 µm.

Aldehydes, including formalin (Formaldehyde CAS # 50-00-0), paraformaldehyde (PFA, CAS # 30525-89-4), and glutaraldehyde (GA, CAS #111-30-8), are commonly used to fix cells, tissues, or media prior to downstream analyses such as histology, immunofluorescence, or electron microscopy. A major hurdle to testing whether aldehyde-treated cells or solutions contain infectious virus is the toxicity of the aldehydes. The addition of aldehyde-containing samples to fresh cells leads to the fixation of the new cells, rendering them incapable of being infected. One method to resolve this issue is dialysis [10], but this can be challenging in the context of containment laboratories, as discussed earlier. The use of size exclusion columns is only suitable for testing small numbers of cells, as the columns easily clog, even with moderate cell numbers. The approach we chose was to wash the fixed cells, which retains virus while being able to remove the fixative, prior to adding the sample to fresh cells, similar to previous reports [12,20]. Here, we present example data, using 10% neutral-buffered formalin to inactivate cells infected with LASV. Our data show the complete inactivation of LASV-infected cells after incubation with formalin for 30 min or 6 h at 4 °C (Figure 5, samples 5 and 6 and Table 2), which is consistent with a previous report showing the formalin inactivation of cells infected with Morogoro virus, a BSL-2 arenavirus used as a surrogate for LASV [13]. Importantly, the negative controls, including mock-infected cells harvested in PBS or formalin (Figure 5, samples 1 and 3), were both negative, and the positive controls, including both LASV-infected cells harvested in PBS and mock-infected, formalin-fixed cells that were challenged with LASV, were positive for LASV (Figure 5, samples 2 and 4). As with the TRIzol testing, the LASV challenge sample was a critical control to show that the addition of PBS-washed formalin-fixed cells did not interfere with infection. Of note, we observed cell clumps for some samples in the test infection flasks, which represent the fixed cells that were added onto healthy cells (Figure 5, Test infection samples). These clumps were not observed after passaging, as only the cell supernatants were subsequently transferred (Figure 5, 1st Passage). Despite the complete inactivation of LASV in infected cells after 30 min of fixation, our approved formalin fixation protocol requires a minimum of 6 h of fixation. This additional required fixation time provides a safety margin to the inactivation procedure. We obtained similar results using 4% PFA for inactivation (Table 2).

We performed similar testing of the ability of 10% formalin and 4% PFA to inactivate EBOV- and NiV-infected cells and 4% PFA to inactivate LASV-infected cells, which also showed complete inactivation at both 30 min and 6 h, consistent with previous reports (Table 2) [10,12,19,23,24,25]. Based on the formalin and PFA inactivation results, we developed an agent- and procedure-specific SOP. The procedure section of this SOP has been added as the Supplementary Materials S2.

In addition to formalin and PFA testing, we provide an example of inactivation testing looking at the ability of GA to inactivate filoviruses. In this case, we used EBOV-expressing ZsGreen (EBOV-ZsG) as our test virus [4]. Using the same fix-then-wash approach as was done for formalin, we showed that 2% GA was able to completely inactivate EBOV-ZsG-infected cells after 1 or 6 h of fixation at 4 °C (Figure S7 and Table 2), consistent with previous results [10,19]. Note that, for EBOV-ZsG-infected cells fixed with GA, there was fluorescence in the test infection flasks (Figure S7, samples 5 and 6). This fluorescence was due to the GA-fixed cells transferred onto these flasks, similar to the cell clumps observed during LASV formalin inactivation testing (Figure 5). Critically, the fluorescence did not spread, was not observed with subsequent transfers of supernatants, and no virus-specific staining was observed.

Another variable to consider when performing inactivation studies is the format of the sample. While the previous described aldehyde samples all involved infected monolayers of cells, other types of samples might be desired for certain assays and would require separate inactivation studies, since the format of the samples could affect the parameters required for effective inactivation. For example, we examined the ability of 10% formalin and 4% PFA to inactivate EBOV-GFP-infected cell pellets. We found that both 10% formalin and 4% PFA were able to inactivate EBOV-GFP under the tested conditions (Table 2). We did not evaluate inactivation of tissue samples, which require longer fixation times than monolayers due to the thickness and density of samples requiring longer times for the fixatives to fully penetrate the samples [10,12,19,23].

We additionally tested the ability of formalin to inactivate EBOV stock solution, which would allow us to perform analyses of virions by electron microscopy. Since mixing formalin with virus stocks would dilute the concentration of formalin in the sample, we utilized 20% formalin and mixed it with an equal volume of concentrated EBOV-ZsG stock (4.6 × 10^8^ _+_TCID_50_ units/mL). However, the fix-then-wash approach would not be appropriate for removing the formalin from these samples, as the virions would be lost using that method. We determined that size exclusion columns were suitable for removing formalin from the samples and had previously determined that these columns retained infectious virus [8]. Using column purification of samples, we found that infectious EBOV-ZsG particles were inactivated when exposed to a final concentration of 10% formalin for 1 or 6 h at 4 °C (Table 2).

One important consideration for inactivating samples with aldehydes is that they can slowly oxidize to acids (e.g., formalin slowly oxidizes to formic acid). This can lead to a reduced fixation capacity of the solution, as well as potentially undesired effects such as the degradation of nucleic acids and the formation of pigment artefacts when exposed to hemoglobin [26,27,28]. The pH of the fixative solution is also particularly important in the context of maintaining ultrastructural elements for electron microscopy [29]. Because of these issues, many fixatives routinely used for immunofluorescence analysis, immunohistochemistry, or similar analyses, such as formalin, are available in a neutral-buffered format. Care should be taken, therefore, when using solutions that lack a pH buffer, such as some commercially available PFA and GA solutions, to make sure that they are used in a timely manner and do not become acidified. For these reasons, our approved aldehyde inactivation SOP requires that fixatives are used within their expiration date if commercially purchased or within 6 months of production for self-diluted buffers.

The fixatives used in this study were chosen based on previous data regarding their ability to inactivate viruses and planned downstream analyses. Faster penetrating aldehydes such as formalin and PFA are typically used for immunofluorescence, immunohistochemistry, or for fixing cells prior to flow cytometry or fluorescence-activated cell sorting analysis. Neutral-buffered formalin is typically preferred for the fixation of animal tissues due to being inexpensive and its longer shelf life, particularly at room temperature. Both GA and mixes of GA with other fixatives (e.g., Karnovsky fixative, which contains both GA and PFA) are often preferred when inactivating samples for electron microscopy, since GA penetrates cells more slowly and better preserves intracellular structures. Conversely, GA is generally not used for fixing cells for immunofluorescence, as it can result in high amounts of background fluorescence as compared to formalin or PFA fixation.

### 3.5. Heat Inactivation

Due to its simplicity, heat treatment is an attractive method for virus inactivation. The advantages of heat inactivation include the lack of chemicals that might interfere with downstream applications, exposure of the entire tube content to heat, which is useful for low-volume samples, and some flexibility regarding the inactivating temperature. However, effective heat inactivation depends heavily on the inactivation conditions, including the sample volume, number of cells, virus load, temperature, and inactivation time. General statements, such as “virus X is inactivated after a 30-min incubation at 60 °C”, are not suitable, because a detailed description of the inactivation conditions is lacking.

Prior to a heat inactivation study, it is essential to ensure that the used equipment—in general, a heat block—reaches the set temperature and that the required temperature is reached in the samples. This can be achieved by using an external thermometer placed in an extra tube filled with water or oil in addition to the internal heat block thermometer. We first measured the temperature in 1.5-mL or 2-mL screw cap tubes filled with 1 mL of water in an Eppendorf Thermomixer set to the maximal temperature of 99 °C. The tube temperature was measured with a temperature sensor inserted through a hole in the lid. Surprisingly, the temperature in the tube remained below 90 °C, significantly lower than the set point and not acceptable for our purposes. We therefore assessed the Advanced Dry Block Heater (VWR) for its suitability for heat inactivation at temperatures above 95 °C. The maximal set temperature for this model is 120 °C. Our data showed that the temperature in the test tubes reliably reached ≥99 °C (100 °C is the boiling point of water) at different positions in the heat block (Figure S8).

**Figure 6:**
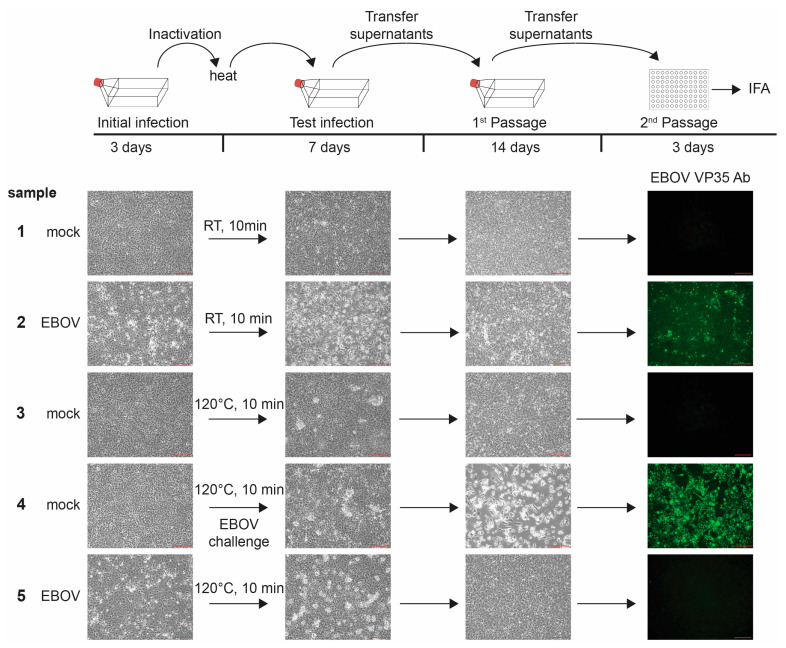
Inactivation of EBOV with heat. Top, schematic of the assay. Vero E6 cells seeded in T175 flasks were mock-infected or infected with EBOV at an MOI of 10. At 3 days post-infection (dpi), brightfield images were taken to assess the presence of CPE in samples as a marker for viral infection (Initial infection). Cells were scraped and transferred into tubes. Samples were incubated at room temperature (RT) or 120 °C and transferred onto Vero E6 cells seeded in T175 flasks. Challenge samples were infected with EBOV at MOI 3. Samples were monitored for CPE at 7 dpi (Test infection). Clarified supernatants were passaged onto Vero E6 cells seeded in T175 flasks. Cells were incubated for an additional 14 days and monitored for viral infection (1st Passage). Clarified supernatants were then used to infect Vero E6 cells seeded in 96-well plates and fixed at 3 dpi. Immunofluorescence analysis (IFA) was performed using an anti-EBOV-VP35 antibody (green, 2nd Passage). Scale bars = 200 µm.

Heat treatment at ≥95 °C, usually in the presence of SDS, is commonly used to inactivate enveloped negative sense RNA viruses to prepare cell lysates for downstream analyses such as Western blot [10,12,13]. We demonstrate that a 10-min incubation at a sample temperature of 99+ °C (block temperature setting: 120 °C) is sufficient to inactivate 1 mL of 2.4 × 10^7^ EBOV-infected cells (Figure 6, sample 5). We were not able to detect live EBOV after this treatment, even after serial passaging and long incubation times (a total of 25 days of incubation). All controls, including the EBOV challenge sample, in which the cells were incubated with heat-treated, uninfected cells prior to infection with EBOV to demonstrate that the cells were still infectable after incubation with heat-treated cells (Figure 6, sample 4), showed the expected results. Similar results were obtained for virus stock inactivation. We were not able to detect live virus in 1 mL of 2 × 10^8^ TCID_50_ units of EBOV particles in 10% FBS treated for 10 min at a sample temperature of 99+ °C (block temperature setting: 120 °C) (Table 2).

Our inactivation studies were performed in the absence of detergents and are in line with previous work that showed that 1 × 10^6^ EBOV-infected cells in a volume of 1 mL were inactivated after 5 and 10 min of incubation, respectively, in a heat block set to 120 °C [10]. In this study, inactivation at a block temperature of 100 °C was observed after 10 min but not after 5 min, emphasizing the importance of well-defined inactivation conditions.

Heat inactivation is also a convenient method for removing cell supernatant samples from the BSL-4 to conduct mycoplasma PCR. We used the QuickExtract™ DNA Extraction Solution in combination with heat inactivation to extract potentially present mycoplasma DNA from cell supernatants or virus stock solutions for PCR analysis. After the samples were heat-inactivated, they were removed from the BSL-4 lab for PCR analysis. The established method includes mixing cell supernatants or virus stocks with QuickExtract™ DNA Extraction Solution, a nontoxic solution that lyses the cells and viral particles. There are two heat steps involved in this procedure. The first heat step for 6 min at 65 °C is required for optimal cell lysis according to the manufacturer’s protocols. Following our standard heat inactivation procedure, we added a second heat step at 120 °C for at least 10 min, which is required for sample inactivation. The extra heat step did not impact the successful detection of mycoplasma DNA (Figure S9A). This easy inactivation method is instrumental for removing BSL-4 samples for routine mycoplasma tests based on DNA PCR (Figure S9B). Of note, adding SDS or other strong detergents to the samples would interfere with polymerase activity.

In summary, heat treatment is a very potent method for inactivating viruses as long as the inactivation conditions are well-defined and rigorous. Our approved heat inactivation protocol requires the use of a reliable heat block set at 120 °C. The inactivation conditions and maximal cell and viral particle numbers are shown in Table 2. An agent- and procedure-specific SOP was developed, and the procedure section is included in the Supplementary Materials S3.

### 3.6. Documentation

Documentation of an inactivation study is a crucial part of the approval process. In addition to describing the purpose of the study, the experimental details, the viral detection methods, the results, and the conclusion, the inactivation report should also cover the LOD study. As an example, we included our report demonstrating the successful inactivation of NiV using TRIzol and TRIzol LS as the Supplementary Materials S4. Inactivation reports are instrumental to reviewers and approvers, including IBCs, biosafety officers, Responsible Officials for Select Agent work, and local or federal regulatory agencies, for evaluating the effectiveness of an inactivation procedure. They also provide the basis for developing and implementing a detailed SOP that ensures that all laboratory workers who are trained on the specific SOP follow the approved inactivation procedure. The procedure sections of some of our SOPs describing inactivation using TRIzol/TRIzol LS, aldehyde fixation, and heat treatment have been added as the Supplementary Materials S1–S3 and can be used as templates for similar documents. It is critical that only inactivated samples are removed from the BSL-4 space. For this reason, our SOPs require that inactivated samples are clearly labeled. In addition, colored lids are used to clearly identify inactivated samples whenever possible.

Since BSL-4 facilities are rare, there is a high demand for collaborations to test antiviral strategies and vaccine candidates targeting highly pathogenic viruses. The infection studies for these collaborations must be performed in a BSL-4 facility, and the materials from these studies must often be inactivated and removed from the BSL-4 laboratory for follow-up analyses. The proper documentation of successful inactivation not only reassures the receivers of the inactivated material that it can be handled safely but is also a requirement for Select Agent work. We put considerable effort into designing an electronic, paperless inactivation certificate for each inactivation procedure that uses dropdown menus and enables electronic signatures. A template for one of our inactivation certificates is provided in the Supplementary Materials S5. The documented information includes the date; inactivation procedure; name of the individual who performed the inactivation procedure; format; virus; checkpoints to ensure that the procedure was performed correctly; signature of the individual who performed the procedure; signature of the responsible Principal Investigator; and removal of the material through the dunk tank for transfer from the BSL-4 laboratory to lower containment with dates, times, and signatures of those who removed it. Using dropdown menus for the names of those who perform the inactivation procedure ensures that only individuals who are approved to perform the respective inactivation procedures are listed. Similarly, dropdown menus for the viruses ensure that the procedure is only applied to viruses for which an approved SOP has been developed. Each inactivation certificate is given a unique identifier, and the form is hosted on a secured electronic drive. Each inactivated sample that is removed from the BSL-4 space is accompanied by the corresponding inactivation certificate that is shared with the receiver of the sample for their records.

## 4. Discussion

In this paper, we present example inactivation methods, including TRIzol/TRIzol LS, aldehyde, and heat inactivation with three different BSL-4 viruses, to illustrate the challenges of each of the inactivation methods and the used viruses. If viruses are inactivated with toxic chemicals, the chemicals must be removed from the inactivated samples to allow for further infection studies in cells. There are different ways to remove toxic chemicals, including dialysis, dilution, or size exclusion column filtration [8,9,10,12,13,17,19,20,22,30]. Irrespective of the used method, however, it is of utmost importance to ensure that the cells treated with the purified inactivated samples are still susceptible to virus infection by including a challenge sample (see Section 3.1, sample 3 in Figure 4, and sample 4 in Figure 5 and Figure 6). The importance of this analysis is highlighted by our results using diluted TRIzol LS. In our hands, although Vero E6 cells treated with diluted TRIzol LS looked unaffected, they were not susceptible to viral infection (Figure S5). Another study, however, showed the successful infection of cells with EBOV following the treatment of cells with TRIzol LS diluted to the same concentration [19]. It is conceivable that the larger format (500 cm^2^ dishes) from the previous study resulted in a lower ratio of TRIzol LS volume per surface area (1.5 µL/cm^2^) compared to our analysis (6-well dishes, 4.7 µL/cm^2^), which might explain the conflicting results. This discrepancy illustrates the importance of including challenge infections as part of an inactivation study.

Multiple studies have shown that TRIzol and TRIzol LS are very well suited to inactivate BSL-4 negative sense RNA viruses [10,12,19,21,22]. In contrast to AVL and RLT buffer inactivation that require the addition of ethanol for complete inactivation [10,18], TRIzol and TRIzol LS alone efficiently kill viruses. In fact, the authors are not aware of a single study that has reported a failed inactivation with TRIzol or TRIzol LS.

Although fixatives are toxic to cells, an easy method to remove them from fixed cells is to rigorously wash the fixed (and inactivated) samples. While this removes the toxic chemicals, adding fixed cells on top of fresh cells to show that the fixed cells do not contain any live virus may lead to confusing results because of a pseudo-CPE appearance caused by the fixed cells that might disguise any real CPE caused by infectious virus (Figure 5). In addition, if recombinant viruses are used that express fluorescent proteins, the fixed, infected cells fluoresce, which makes it difficult to discriminate between fixed cells and newly infected cells (Figure S7). An easy way to overcome this issue is to passage the cell supernatants one to two times, which clarifies the samples from the fixed material and leads to clear results regarding CPE and infection with both wildtype and fluorescent viruses (Figure 5 and Figure S7). Similar to TRIzol, aldehydes reliably inactivate negative sense RNA viruses under the conditions tested here (10% formalin, 4%PFA, or 2% GA incubated on cell monolayers for 6 h at 4 °C). This is in line with other studies on BSL-4 viruses that uniformly showed inactivation under similar conditions [10,12,13,19,23,24,25].

Physical inactivation, such as heat, comes with a different set of challenges compared to chemical inactivation. There have been many reports of failed heat inactivation, specifically for temperatures below 60 °C. In a previous study, the incubation of EBOV stocks for one hour at 56 °C resulted in a biphasic inactivation curve with a steep decline in viral titers in the first 20 min and a much slower inactivation profile for a second, more heat-resistant virus population [24]. The nature of this heat-resistant virus population is not known, but it is conceivable that aggregated viral particles may play a role. Based on the inactivation curves, which showed a considerable amount of infectious virus after 30 min of incubation at 56 °C, Bowen and colleagues recommended 60 min of incubation at 60 °C for complete inactivation [24]. This was supported by a comparative thermal inactivation study on EBOV, Marburg virus, and LASV, which showed the complete inactivation of 5 × 10^4^ to 5 × 10^6^ plaque-forming units (PFU) added to human serum in a total volume of 0.5 mL after 60 min of incubation at 60 °C but not at 45 °C or 56 °C [31]. Similarly, Olschewski and colleagues also observed the complete inactivation of Morogoro arenavirus, a surrogate for LASV, after 60 min of incubation at 60 °C [13].

We previously demonstrated the complete inactivation of infectious EBOV in both low-volume (0.1 mL, 1.67 × 10^6^ TCID_50_ units) and high-volume (1 mL, 1.67 × 10^7^ TCID_50_ units) samples with 60 min of incubation at 60 °C. When the incubation time was reduced to 30 min at 60 °C, we still observed complete inactivation of the low-volume sample, whereas the high-volume samples still contained infectious virus [8]. In line with our results, Haddock and colleagues reported the incomplete inactivation of 1 × 10^6^ EBOV particles in a total volume of 1 mL when incubated for 30 min at 60 °C or 65 °C, respectively [10], emphasizing the sample volume as a crucial factor in heat inactivation. In contrast, a recent study by Widerspick and colleagues on NiV inactivation showed the complete inactivation of 1 mL of NiV supernatants containing 1 × 10^6^ PFU after 30 min of incubation at 56 °C [12]. Another NiV study reported a 4 log_10_ reduction in viral titers when 200 µL of NiV stocks containing 6 × 10^5^ TCID_50_ units were treated for 30 min at 56 °C. These samples still contained a low amount of live virus, whereas viral titers were below the detection limit when the samples were treated for 60 min at 56 °C or 60 °C, respectively [32]. A study by Chmielewski and colleagues on the inactivation of avian viruses, including Newcastle disease virus (NDV), showed that a 6 log_10_ reduction in NDV titers required at least 10 min at 59 °C, 5 min at 70 °C, and less than 30 s at 80 °C [33]. Taken together, the data generated for different viruses suggest that, to a certain degree, longer incubation times compensate for lower inactivation temperatures. However, there are limitations, and temperatures below 60 °C have been identified as unreliable in multiple studies. As a rule of thumb, the data presented here, along with published works, suggest a strategy of the-hotter-the-better for virus inactivation. While BSL-4 negative sense RNA viruses are reliably inactivated at temperatures at or above 95 °C, as shown by us (Table 1) and others [10], high temperatures may interfere with downstream applications, such as serology. However, reducing the temperature for inactivation requires careful validation of the inactivation conditions.

As a safety margin, many heat inactivation procedures include the addition of a detergent, usually 1–2% sodium dodecyl sulfate (SDS), prior to incubation of10 min at 95 °C [10,12,13]. SDS not only inactivates EBOV in the absence of heat [10], it also elevates the boiling point of a watery solution, increasing the temperature in the tubes. To confirm the SDS-mediated boiling point elevation, we measured the temperature in 2 mL tubes filled with 1 mL of PBS + 1% SDS or radioimmunoprecipitation assay (RIPA) buffer (1% Nonidet P-40 and 0.1% SDS). RIPA buffer is commonly used for cell lysis. As expected, the temperature in all tubes was above 100 °C (data not shown). However, there is an advantage in performing a heat inactivation study in the absence of chemicals, because it allows more flexibility in the choice of the added reagent. While the combination of ≥95 °C and SDS is commonly used to prepare BSL-4 cell lysates for Western blot analysis [34,35], the addition of SDS is prohibitive for other analyses downstream of cell lysis, such as proteomics analyses. Other detergents or chaotropic agents, such as lithium dodecyl sulfate (LDS) or guanidinium hydrochloride (GuHCl), may contribute to virus inactivation [30] but do not interfere with proteomics or RNA–protein interaction studies [4,36].

In conclusion, our work shows that inactivation methods commonly used to inactivate BSL-4 negative sense RNA viruses are highly reliable and reproducible across viral families. However, it is important to precisely define the inactivation parameters and to include controls to ensure that the inactivation validation procedure overcomes potential challenges such as removing toxic components from the samples. Defining the exact inactivation parameters is particularly important for heat inactivation. We are confident that the provided SOP information, inactivation report, and sample inactivation certificate will be useful for other labs conducting similar studies.

## Figures and Tables

**Table 1:** Results of the limit of detection (LOD) analysis. Positive replicates were determined by CPE, virus-specific immunofluorescence analysis, and fluorescence for GFP- and ZsGreen-expressing viruses.

Virus	Limit of Detection (TCID_50_ Units)	Number of Positive Replicates/Total Number of Replicates
EBOV	1	2/4
	10	2/4
	100	3/3
EBOV-GFP	1	3/3
	10	3/3
EBOV-ZsGreen	1	3/4
	10	4/4
NIV	1	2/4
	10	4/4
	100	2/2
LASV	1	2/4
	10	4/4
	100	2/2

**Table 2:** Inactivation methods approved for BSL-4 viruses in the NEIDL. * Validated inactivation method but not approved as an inactivation procedure to remove samples from BSL-4. To increase the safety margin for inactivated samples, a 6-h treatment with aldehydes is used as an approved inactivation procedure. ** Although heat (10 min, >99 °C) is a validated inactivation method, a second step such as the addition of detergents (e.g., SDS and LDS) or GuHCl is required for the approved inactivation procedure as an added safety margin. *** Each inactivation method alone is sufficient to inactivate the samples, although both are required for the approved inactivation procedure [8].

Inactivation Method	Inactivation Conditions	Sample Type	Maximum Amounts	Virus Tested
TRIzol	10 min, RT	cells	2.0 × 10^7^ cells	EBOV
			2.7 × 10^7^ cells	NiV
			2.8 × 10^7^ cells	LASV
				
TRIzol LS	10 min, RT	viral particles	2.25 × 10^8^ TCID_50_ units	EBOV
			1.25 × 10^7^ TCID_50_ units	NiV
			9.9 × 10^7^ TCID_50_ units	LASV
				
10% formalin or 4% PFA	30 min, 4 °C * 6 h, 4 °C	adherent cells	1.7 × 10^7^ cells	EBOV
			2.7 × 10^7^ cells	NiV
			2.6 × 10^7^ cells	LASV
				
10% formalin or 4% PFA	6 h, 4 °C	cell pellets	9 × 10^6^ cells	EBOV-GFP
				
10% formalin	1 h, 4 °C * 6 h, 4 °C	viral particles	2.3 × 10^8^ TCID_50_ units	EBOV-ZsGreen
				
2% glutaraldehyde	1 h, 4 °C * 6 h, 4 °C	adherent cells	4.0 × 10^6^ cells	EBOV-ZsGreen
				
heat	10 min, >99 °C **	cells	2.4 × 10^7^ cells	EBOV
				
heat	10 min, >99 °C **	viral particles	2.25 × 10^8^ TCID_50_ units	EBOV
				
TCL combined with heat	10 min, RT (TCL) *** 45 min, 60 °C ***	cells	3.7 × 10^5^ cells	EBOV

## Data Availability

Data are contained within the article or Supplementary Materials.

## Supplementary Materials

The following supporting information can be downloaded at: https://www.mdpi.com/article/10.3390/pathogens12070952/s1: Supplementary Materials S1: Procedure section of SOP inactivation BSL-4 material using TRIzol or TRIzol LS. Supplementary Materials S2: Procedure section of SOP inactivating BSL-4 material using aldehydes. Supplementary Materials S3: Procedure section of SOP inactivating BSL-4 material using heat. Supplementary Materials S4: Report on the inactivation of NiV by TRIzol and TRIzol LS. Supplementary Materials S5: Sample inactivation certificate: Certificate of inactivation of BSL-4 materials using TRIzol or TRIzol LS. Figure S1: Schematic of TRIzol and TRIzol LS inactivation testing. Figure S2: Schematic of aldehyde inactivation testing. Figure S3 Schematic of heat inactivation testing. Figure S4: Limit of detection (LOD) analysis. Figure S5: Cells pretreated with diluted TRIzol LS appear normal but are not infectable. Figure S6: Virus recovery from Amicon columns. Figure S7: Inactivation of EBOV-ZsGreen with 2% glutaraldehyde. Figure S8: Validation of VWR Advanced Dry Block heater. Figure S9: Validation of mycoplasma detection kit. Table S1: Virus-specific conditions of inactivation testing. References [37,38,39] are cited in the supplementary materials.
