# Supplementary material for: Art of the Kill: Designing and Testing Viral Inactivation Procedures for Highly Pathogenic Negative Sense RNA Viruses
Source: Pathogens. 2023 Jul 19;12(7):952. doi: 10.3390/pathogens12070952 (PMC10386221; doi:10.3390/pathogens12070952)
Supplement: Supplementary file 1 [file pathogens-12-00952-s001.zip › Supplementary figures revised 2.pdf]

Art of the kill: designing and testing viral inactivation procedures for highly pathogenic negative sense RNA viruses

Judith Olejnik [1,2,ǂ], Adam J. Hume [1,2,ǂ], Stephen J Ross [1,2,3], Whitney A. Scoon [1,2], Scott Seitz [1,2], Mitchell R. White [1,2], Ben Slutzky [2], Nadezhda E Yun [2], and Elke Mühlberger [1,2,*]

1 Department of Virology, Immunology and Microbiology, Chobanian & Avedisian School of Medicine, Boston University, Boston, MA 02118
2 National Emerging Infectious Diseases Laboratories, Boston University, Boston, MA 02218
3 Department of Biochemistry and Cell Biology, Chobanian & Avedisian School of Medicine, Boston University, Boston, MA 02118

4 ǂ Equal contribution
* Correspondence: muehlber@bu.edu, Tel.: +1 617-358-9153

Figures S1-9, Table S1, Supplementary Material S1-5

Citation: To be added by editorial staff during production.

Academic Editor: Firstname Lastname

Received: date
Revised: date
Accepted: date
Published: date

Copyright: © 2023 by the authors. Submitted for possible open access publication under the terms and conditions of the Creative Commons Attribution (CC BY) license (https://creativecommons.org/licenses/by/4.0/).

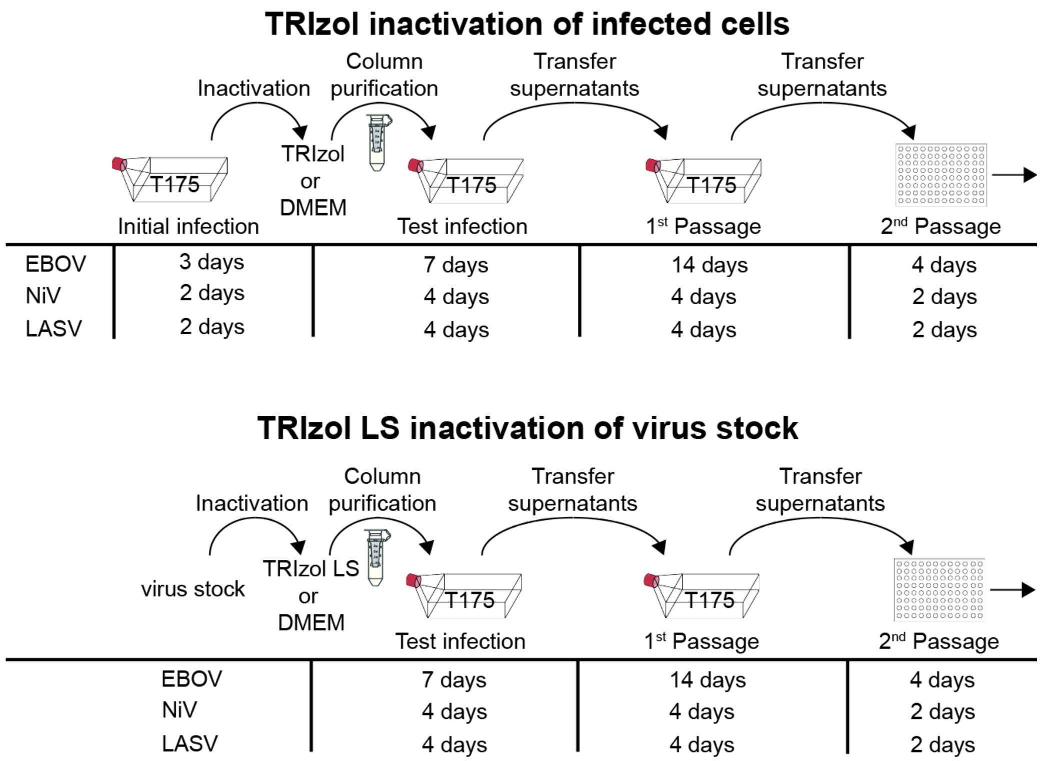

Figure S1. Schematic of TRIzol and TRIzol-LS inactivation testing.

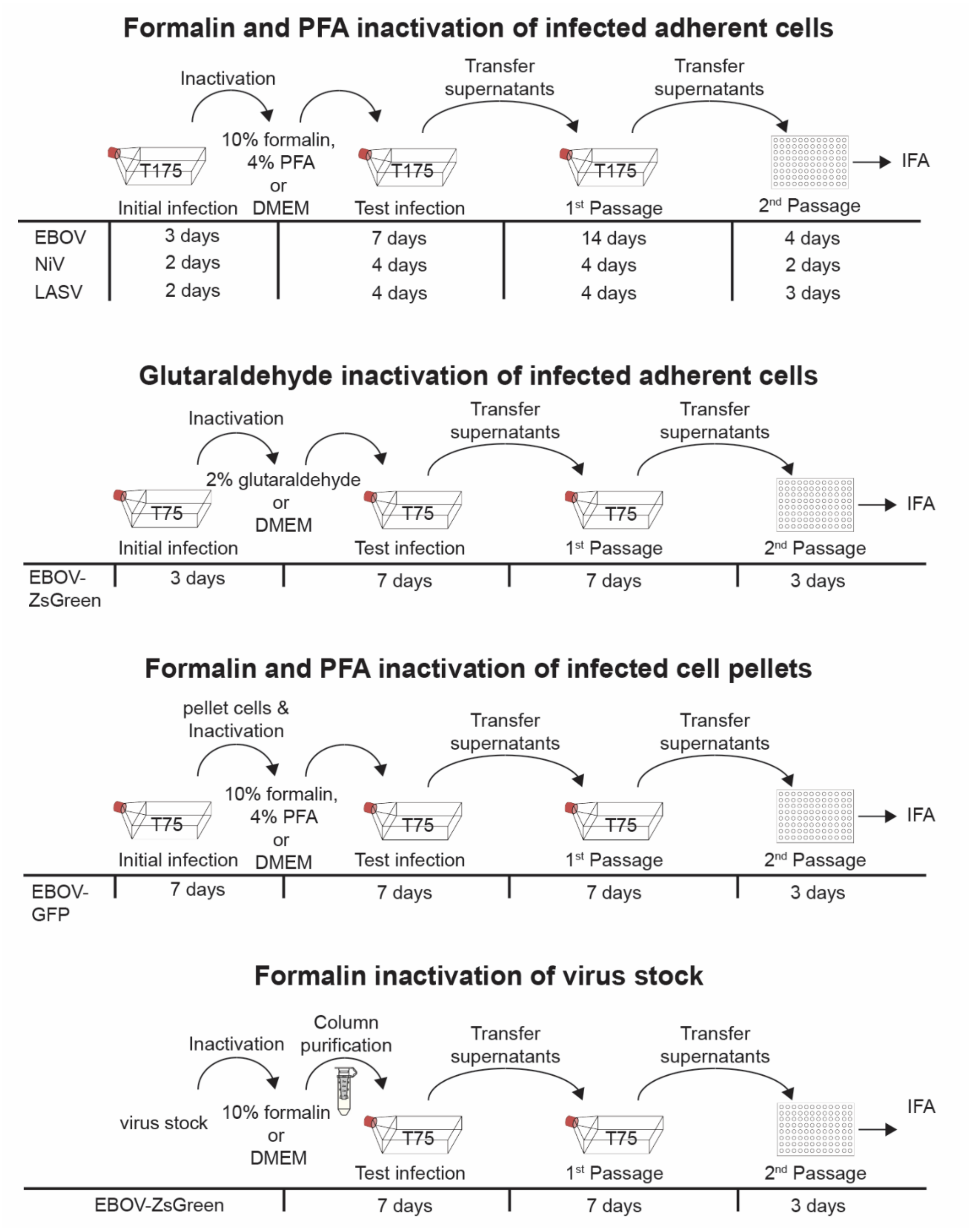

Figure S2. Schematic of aldehyde inactivation testing.

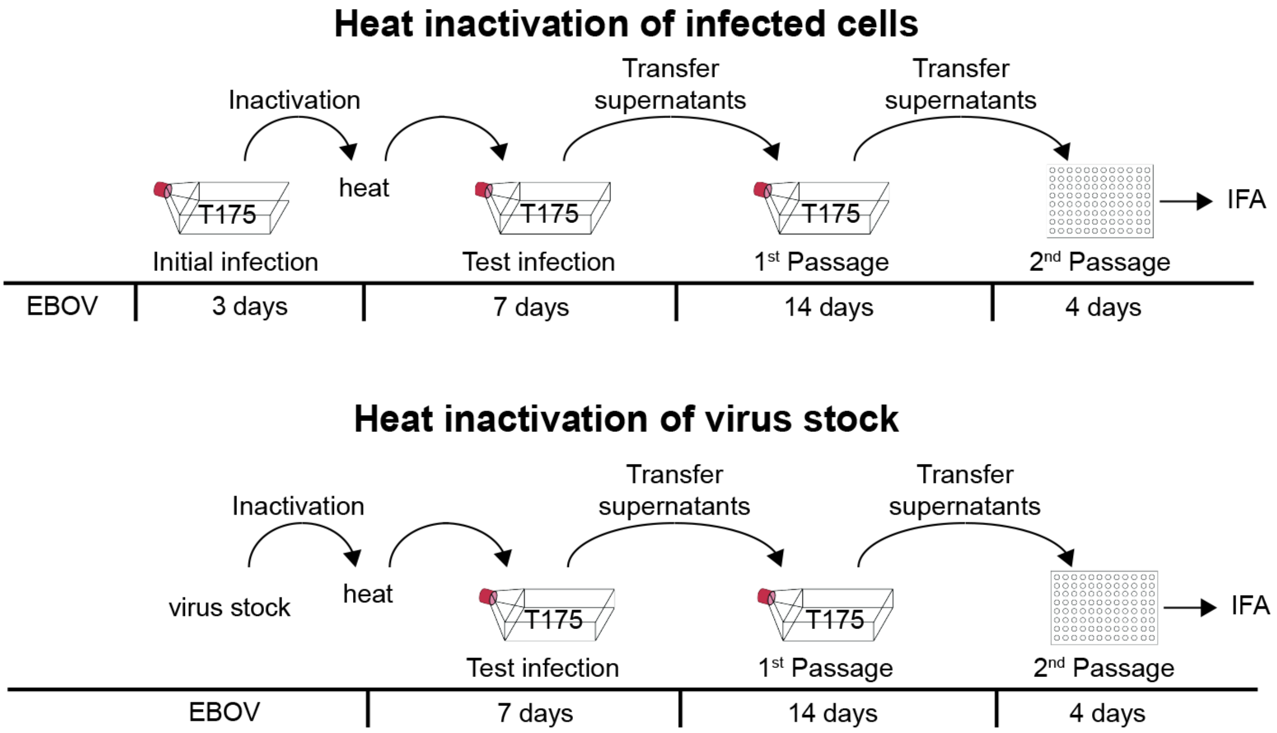

Figure S3. Schematic of heat inactivation testing.

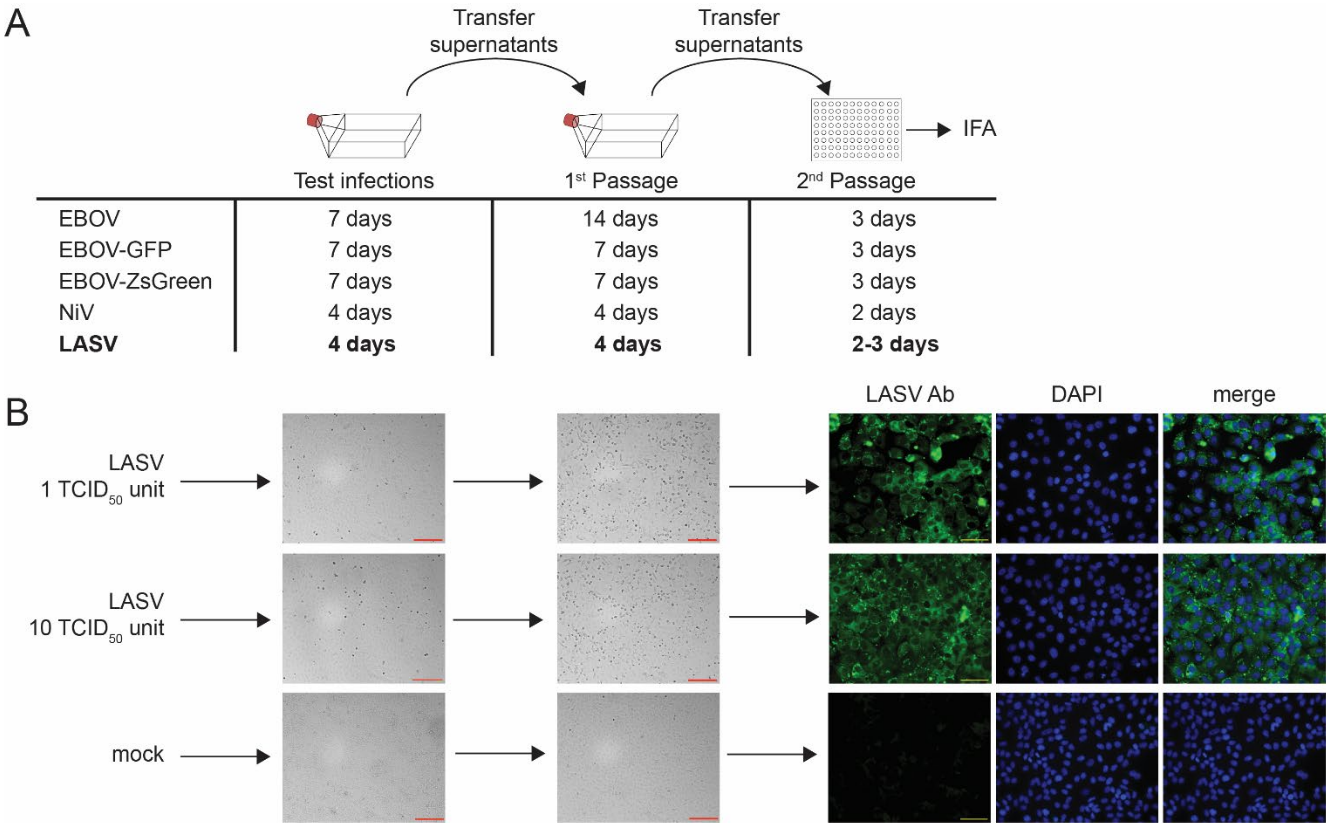

Figure S4. Limit of detection (LOD) analysis. (A) Schematic of LOD analysis. LASV is highlighted because it was used as an example in (B). (B) LASV LOD analysis. Vero E6 cells seeded in T175 flasks were mock infected or infected with LASV with the indicated $TCID_{50}$ units. Samples were monitored for CPE at 4 dpi. Clarified supernatants were passaged onto Vero E6 cells seeded in T175 flasks. Cells were incubated for additional 4 days and monitored for viral infection. Clarified supernatants were then used to infect Vero E6 cells seeded in 96-well plates and fixed at 2 dpi. Immunofluorescence analysis was performed using an anti-LASV antibody (LASV ab, green). Cell nuclei were stained with DAPI (blue). Red scale bars = 200 μm; yellow scale bars = 50 μm.

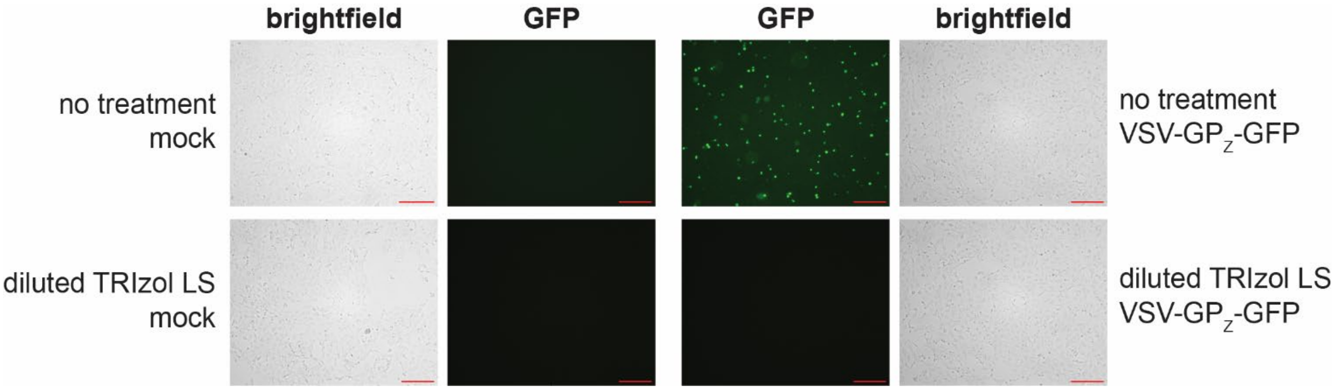

Figure S5. Cells pre-treated with diluted TRIzol LS appear normal but are not infectable. Vero E6 cells seeded in a 6-well dish were either not treated or treated with diluted TRIzol LS (45 μL in 3 mL media). Cells were then mock infected or infected with recombinant vesicular stomatitis virus (VSV) expressing EBOV GP in place of VSV G and expressing GFP as an additional open reading frame (VSV-GP$_Z$-GFP) at an MOI of 0.1. Samples were monitored for CPE and GFP expression at 2 dpi. Red scale bars = 200 μm.

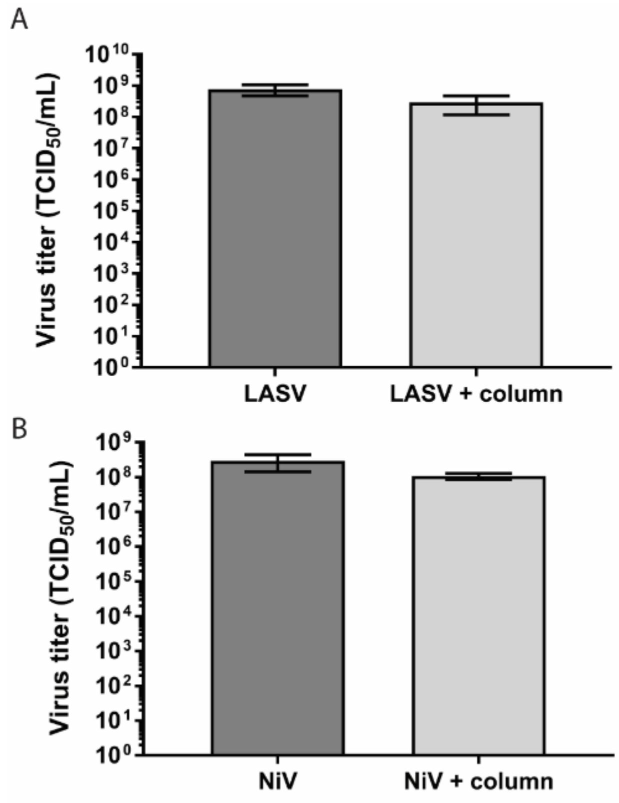

Figure S6. Virus recovery from Amicon columns. 0.5 mL NiV stock containing 2.5×10^8 $TCID_{50}$ units or 0.5 mL LASV stock containing 2×10^8 $TCID_{50}$ units were added to size exclusion columns. Column purification was performed according to the manufacturer's instructions. Viral titers of samples eluted from the column and non-column purified virus stocks were determined by $TCID_{50}$ assay.

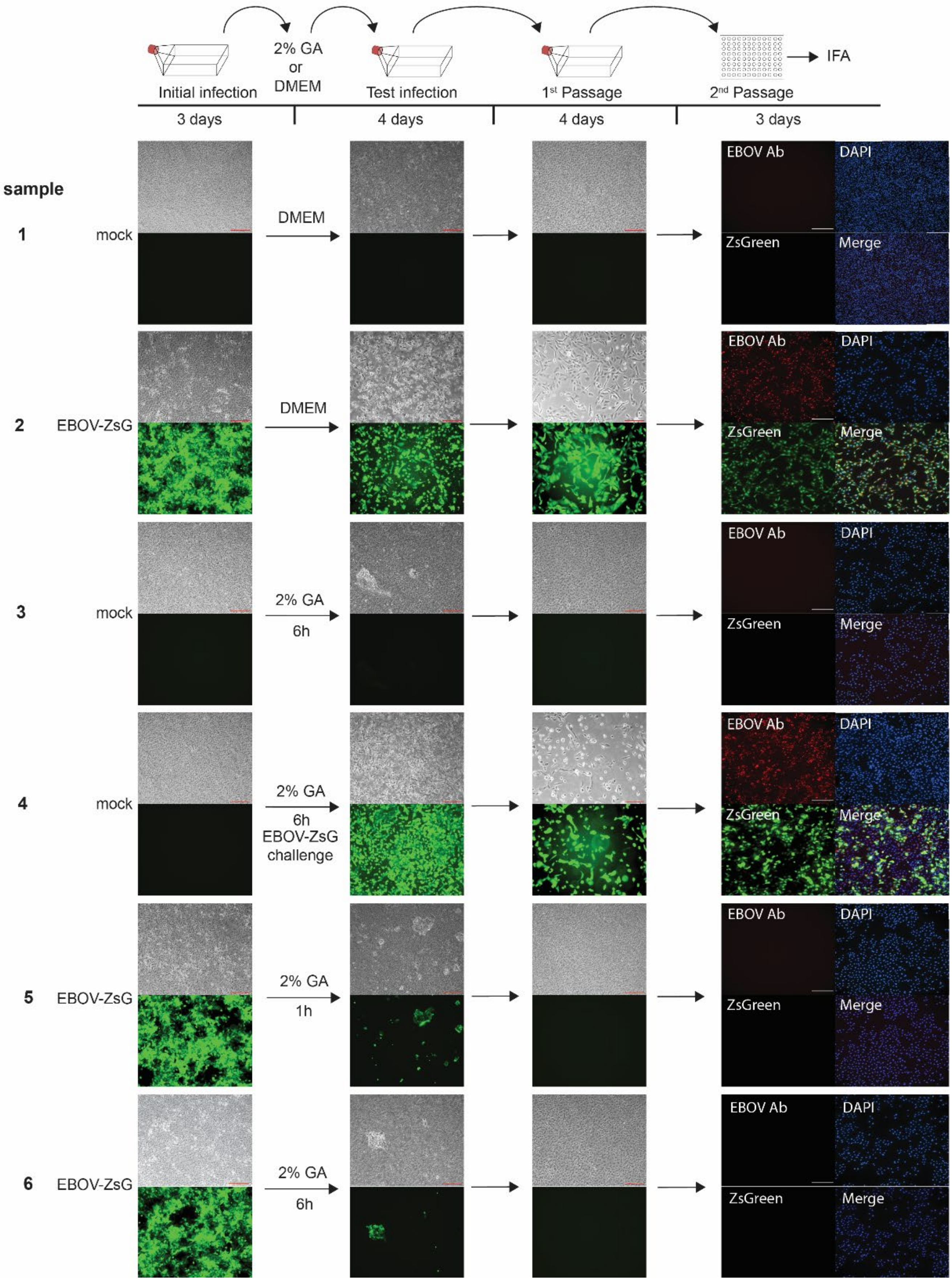

Initial infection
2% GA
or
DMEM
Test infection
1st Passage
2nd Passage
IFA
3 days
4 days
4 days
3 days
sample
1
mock
DMEM
2
EBOV-ZsG
DMEM
3
mock
2% GA
6h
4
mock
2% GA
6h
EBOV-ZsG
challenge
5
EBOV-ZsG
2% GA
1h
6
EBOV-ZsG
2% GA
6h
EBOV Ab
DAPI
ZsGreen
Merge

Figure S7. Inactivation of EBOV-ZsGreen with 2% glutaraldehyde. Top, schematic of the assay. Vero E6 cells seeded in T75 flasks were mock infected or infected with EBOV-ZsGreen (EBOV-ZsG) at an MOI of 3. At 3 days post infection (dpi), brightfield images were taken to assess the presence of cytopathic effect (CPE) and green fluorescence (ZsGreen) in samples as a marker for viral infection (Initial infection). Cells were fixed in 2% glutaraldehyde (GA) or incubated in PBS, scraped, washed with PBS and transferred onto Vero E6 cells seeded in T75 flasks. Challenge samples were infected with EBOV-ZsGreen at MOI 3. Samples were monitored for CPE and fluorescence at 4 dpi (Test infections). Clarified supernatants were passaged onto Vero E6 cells seeded in T75 flasks. Cells were incubated for additional 4 days and monitored for viral infection (1st Passage). Clarified supernatants were then used to infect Vero E6 cells seeded in 96-well plates and fixed at 2 dpi. Immunofluorescence analysis (IFA) was performed using an anti-EBOV-VP35 antibody (red, 2nd Passage). Cell nuclei were stained with DAPI (blue). Red scale bars = 200 μm; white scale bars = 250 μm.

A

Block I

	1	2	3	4	5
a	123.2°C	123.8°C	123.6°C	123.1°C	122.8°C
b	123.3°C	123.9°C	123.6°C	122.7°C	123.1°C
c	123.2°C	123.5°C	122.0°C	122.3°C	123.0°C
d	122.9°C	123.8°C	122.6°C	122.1°C	121.8°C

Block II

123.8°C	123.5°C	123.7°C	123.4°C	123.1°C
123.1°C	122.9°C	122.7°C	121.4°C	123.1°C
121.4°C	123.2°C	122.5°C	121.9°C	121.8°C
122.9°C	122.1°C	122.2°C	121.6°C	123.0°C

B

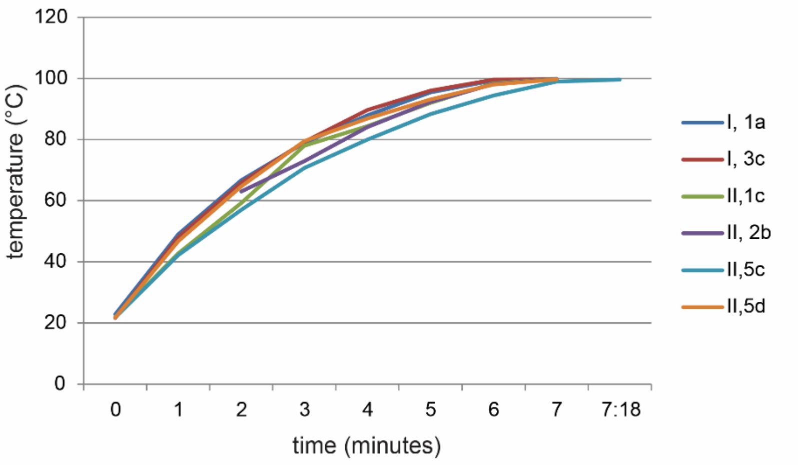

Figure S8. Validation of VWR Advanced Dry Block Heater. (A) The block heater was set at 120°C. When the temperature of 120°C was reached as indicated by the internal block thermometer, the temperature in the wells of the block heater was measured with an external thermometer (Monacor DTM-506RS digital temperature sensor). (B) The block heater was set at 120°C. 2 mL screw cap tubes were filled with 1 mL H_2O and placed in the wells highlighted in blue. The temperature in the tubes was recorded over time using a Monacor DTM-506RS digital temperature sensor.

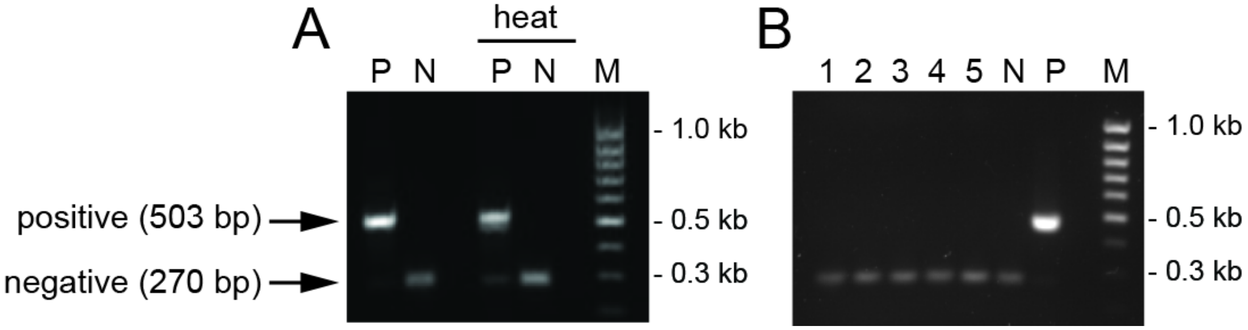

Figure S9. Validation of the Mycoplasma Detection kit when an additional heat inactivation step was implemented. (A) The Mycoplasma Detection kit from Southern Biotech was used according to the manufacturer's instructions. M. orale DNA is included in the kit as a positive control and leads to a 503 base pair (bp) PCR fragment. An internal negative control (270 bp) is included to prevent false negatives due to PCR inhibitors. Detection of correct sized bands is shown after additional heat treatment for inactivation of samples. P, positive control; N, negative control; heat, additional heat inactivation step (at least 10 minutes at 120°C); M, DNA size marker. (B) Example of BSL-4 virus stock mycoplasma testing (samples 1-5) based on PCR and using an additional heat inactivation step (at least 10 minutes at 120°C).

Inactivation method	Sample type	Virus tested	MOI used for initial infection or virus amount tested	MOI used for challenge infection
TRIzol	cells	EBOV	MOI 10	MOI 3
		NiV	MOI 0.5	MOI 0.01
		LASV	MOI 5	MOI 0.1
TRIzol-LS	viral particles	EBOV	$2x10^8$ $TCID_{50}$ units	MOI 3
		NiV	$1.25x10^7$ $TCID_{50}$ units	MOI 0.01
		LASV	$9.9x10^7$ $TCID_{50}$ units	MOI 0.1
10% formalin or 4% PFA	adherent cells	EBOV	MOI 10	MOI 3
		NiV	MOI 0.5	MOI 0.01
		LASV	MOI 5	MOI 0.1
10% formalin or 4% PFA	cell pellets	EBOV-GFP	MOI 5	MOI 5
10% formalin	viral particles	EBOV-ZsGreen	$2.3x10^8$ $TCID_{50}$ units	MOI 3
2% glutaraldehyde	adherent cells	EBOV-ZsGreen	MOI 3	MOI 3
heat	cells	EBOV	MOI 10	MOI 3
	viral particles		$2x10^8$ $TCID_{50}$ units	MOI 3
TCL combined with heat	cells	EBOV	MOI 10	MOI 3

Table S1. Virus-specific conditions for the performed inactivation studies.
