# Supplementary material for: Art of the Kill: Designing and Testing Viral Inactivation Procedures for Highly Pathogenic Negative Sense RNA Viruses
Source: Pathogens. 2023 Jul 19;12(7):952. doi: 10.3390/pathogens12070952 (PMC10386221; doi:10.3390/pathogens12070952)
Supplement: Supplementary file 1 [file pathogens-12-00952-s001.zip › Supplementary Materials S1-5 updated.pdf]

Art of the kill: designing and testing viral inactivation procedures for highly pathogenic negative sense RNA viruses

Judith Olejnik [1,2,ǂ], Adam J. Hume [1,2,ǂ], Stephen J Ross [1,2,3], Whitney A. Scoon [1,2], Scott Seitz [1,2], Mitchell R. White [1,2], Ben Slutzky [2], Nadezhda E Yun [2], and Elke Mühlberger [1,2,*]

1 Department of Virology, Immunology and Microbiology, Chobanian & Avedisian School of Medicine, Boston University, Boston, MA 02118
2 National Emerging Infectious Diseases Laboratories, Boston University, Boston, MA 02218
3 Department of Biochemistry and Cell Biology, Chobanian & Avedisian School of Medicine, Boston University, Boston, MA 02118

4 ǂ Equal contribution
* Correspondence: muehlber@bu.edu, Tel.: +1 617-358-9153

Supplementary Materials S1-5

Citation: To be added by editorial staff during production.

Academic Editor: Firstname Lastname

Received: date
Revised: date
Accepted: date
Published: date

Copyright: © 2023 by the authors. Submitted for possible open access publication under the terms and conditions of the Creative Commons Attribution (CC BY) license (https://creativecommons.org/licenses/by/4.0/).

Supplementary Material S1

Procedure section of SOP *Inactivating BSL-4 material Using TRIzol or TRIzol LS*

This SOP does only contain the procedure section. It does not contain roles and responsibilities, required equipment and supplies, training requirements, safety requirements, personal protective equipment etc. The indicated links are deactivated.

1. Procedures and Instructions

The procedures described in this SOP are approved for filoviruses, paramyxoviruses and arenaviruses. TRIzol and TRIzol LS used for inactivation must be maintained according to manufacturer's specifications and must be within the expiration date.

All work with infectious material must be performed in a biological safety cabinet following SOP, Use of the Class II Biological Safety Cabinet. Procedures for centrifugation will conform with the SOP corresponding to the centrifuge used. Removing inactivated samples will conform to SOP, Removal of Non-viable Material from BSL-4 Containment Space.

Approved inactivation methods

Type of Inactivation	Sample type	Maximal cell number/ maximal virus amount		
**TRIzol**	Cells (monolayer, suspension)	up to $2x10^7$ cells		
**TRIzol LS**	Cell lysates	up to $2x10^7$ cells in a minimal volume of 1 mL of cell lysis buffer		
**TRIzol LS**	Supernatant, virus stock (max 10% FBS)	**Filovirus**	**Paramyxovirus**	**Arenavirus**
		Up to $2.25x10^8$ $TCID_{50}$ units in a minimal volume of 250 µL	Up to $1.25x10^7$ $TCID_{50}$ units in a minimal volume of 250 µL	Up to $9.9x10^7$ $TCID_{50}$ units in a minimal volume of 250 µL

Table 1. Approved inactivation parameters. All approved sample types are listed in the table. Any sample outside these parameters must be brought to the attention of EHS and cannot be removed from BSL-4 containment. Non-SA coronaviruses, coronaviruses that are not classified as select agents.

Inactivation of cells infected with filoviruses, paramyxoviruses, arenaviruses or non-select agents with TRIzol:

1.1. **Monolayers:** Remove supernatant from the cells and add a minimum volume of 1 mL of TRIzol per 10 cm^2 of cell culture dish surface area (see table 2 and Appendix I, *Surface areas of cell culture vessels and cell numbers*). Lyse cells completely by pipetting and transfer lysates into screw cap tubes. Avoid splashing. Proceed with 1.3.

1.2. **Suspension/detached/scraped cells:** Alternatively, scrape cells directly into the supernatant and transfer cell-containing supernatant into an appropriate tube. Spin cells down according to SOP, Use of Low-Speed Centrifuges with Biohazardous Agents in BSL-4 Laboratories or SOP; Use of Microcentrifuges with Biohazardous Agents, depending on the centrifuge used. Follow the same procedure for suspension cells. Remove supernatant and re-suspend cell pellet thoroughly in a minimum volume of 1 mL of TRIzol for up to $2x10^7$ cells (see Table 2 and Appendix I, *Surface areas of cell culture vessels and cell numbers*).

1.3. Vortex samples and incubate at room temperature for 10 minutes for virus inactivation.

1.4. Transfer the entire contents of the tube to a new tube.

1.5. Apply TRIzol reagent over the thread of the lid, doping the thread.

1.6. Use a colored lid to close tube, which indicates that the tube contains an inactivated sample. Vortex tube vigorously.

1.7. Complete form Certificate of Inactivation of BSL-4 materials by TRIzol or TRIzol LS on Sharepoint (Inactivation Certificates).

1.8. Remove the inactivated samples from the BSL-4 laboratory, following SOP, Removal of Non-viable Material from BSL-4 Containment Space.

Cell culture vessel	Cell numbers	Minimum volume of TRIZOL (mL)
Suspension cells	up to $2x10^7$ cells	1 mL
Well of 6 well plate	approx. $1.2x10^6$ cells	1 mL
Well of 12 well plate	approx. $5.0x10^5$ cells	0.75 mL
Well of 24 well plate	approx. $2.5x10^5$ cells	0.5 mL
Well of 96 well plate	approx. $5x10^4$ cells	0.2 mL

Table 2: Recommended volumes of TRIzol to be used for inactivation for common cell culture vessels

Inactivation of supernatants containing filoviruses, paramyxoviruses, arenaviruses or non-select agents with TRIzol LS:

1.9. Clarify cell supernatants by low-speed centrifugation and transfer them into fresh tubes.

1.10. Add 0.75 ml TRIzol LS per 0.25 ml of sample volume**. Note that a 3:1 ratio of TRIzol LS reagent to sample volume is critical for effective inactivation.**

1.11. Vortex samples and incubate for 10 min at room temperature for virus inactivation.

1.12. Transfer the entire contents of the tube to a new tube.

1.13. Apply TRIzol LS reagent over the thread of the lid, doping the thread.

1.14. Use a colored lid to close tube, which indicates that the tube contains an inactivated sample. Vortex tube vigorously.

1.15. Complete electronic form *TRIzol/TRIzol LS Inactivation Certificate* on Sharepoint (Inactivation Certificates).

1.16. Remove the inactivated samples from the BSL-4 laboratory, following SOP, Removal of Non-viable Material from BSL-4 Containment Space.

2. Forms

Certificate of Inactivation of BSL-4 materials using TRIzol or TRIzol LS (Inactivation Certificates).

3. Records Management

Correct performance of this SOP and removal of TRIzol- or TRIzol LS-inactivated samples from BSL-4 containment must be documented by completing electronic form *TRIzol/TRIzol LS Inactivation Certificate* on Sharepoint.

Supplementary Material S2

Procedure section of SOP *Inactivating BSL-4 material using aldehydes*

This SOP does only contain the procedure section. It does not contain roles and responsibilities, required equipment and supplies, training requirements, safety requirements, personal protective equipment etc. The indicated links are deactivated.

1. Procedures and Instructions

The procedures described in this SOP are approved for filoviruses. Inactivation of infected cells grown in a monolayer with 10% formalin or 4% PFA is also approved for paramyxoviruses (henipaviruses) and arenaviruses as indicated in **Table 1**. Commercially purchased 10% formalin, 20% formalin, or 2% Glutaraldehyde (GTA) has to be used by the expiration date. SOP In-house Preparation of Aldehyde Based Fixatives is used to freshly prepare 4% PFA or 2% GTA, which has to be stored at 4°C and used within 6 months.

Work to be performed in the biosafety cabinet will conform to SOP, Use of the Class II Biological Safety Cabinet.

Approved inactivation methods for infected cells

Type of Inactivation	Virus	Sample type	Minimum volume 10% formalin, 4% PFA, or 2% GTA	Maximal number of infected cells
10% formalin or 4% PFA	filoviruses	Monolayer	200 µL/cm^2 surface area	up to 1.6 x10^7 cells
10% formalin or 4% PFA	paramyxoviruses	Monolayer	200 µL/cm^2 surface area	up to 2.7 x10^7 cells
10% formalin or 4% PFA	arenaviruses	Monolayer	200 µL/cm^2 surface area	up to 2.6 x10^7 cells
10% formalin or 4% PFA	filoviruses	Cell pellet	2 mL	up to 9 x10^6 cells
2% GTA	filoviruses	Monolayer	200 µL/cm^2 surface area	up to 1 x10^7 cells

Table1. Approved inactivation parameters for cells. All approved sample types are listed in the table. Any sample outside these parameters must be brought to the attention of EHS and cannot be removed from BSL-4 containment.

Approved inactivation methods for viral particles

Type of Inactivation	Virus	Sample type	Minimum volume 20% formalin	Maximal number of viral particles (filoviruses)
10% formalin	filoviruses	Viral particles	100 µL (total volume: 200 µL)	2.3x10^8 TCID50 units

Table 2. Approved inactivation parameters for viral particles. All approved sample types are listed in the table. Any sample outside these parameters must be brought to the attention of EHS and cannot be removed from BSL-4 containment.

Special precautions for the use of glass: The use of glass in the BSL-4 space is strongly discouraged. In case there are no other alternatives to the use of glass, EHS has approved the use of glass coverslips and slides for this SOP. Extreme caution must be exercised when handling glass. All staff performing this procedure must provide documentation of glass handling training.

When glass coverslips are used, each coverslip must be accounted for at all times. Sharp containers must be easily accessible and located as close as feasible to the immediate area where glass coverslips or glass slides are used. The loss of glass coverslips must be immediately reported to the Core Director or Associate Director and other BSL-4 users. Uncontained glass coverslips in the biosafety cabinet or in the sink can cause glove damage and must be treated as sharps. Glass coverslips must be handled with tweezers; handle with caution.

When using glass chamber slides, do not separate the glass slide from the plastic chamber within the BSL-4 laboratory.
When working with glass coverslips or glass slides, place paper towels or absorbent sheets over the work area. If the coverslip/slide falls or breaks during the experiment, the paper towels or sheets will be used to discard broken glass into a sharps container (with the paper towels/sheets).

1.1. **Coverslips**: Transport cell culture plate(s) containing the coverslips to BSC (SOP, Use of the Class II Biological Safety Cabinet) and remove supernatant. Optional: wash cells with an appropriate washing buffer (e.g. PBS) to remove residual cell culture media. Each well of the cell culture plate must be completely filled with fixative, including those which were not used during the experiment. Alternatively, transfer the coverslips into a new cell culture plate and fill wells with 10% formalin, 4% PFA, or 2% GTA per well. Place plate(s) into a leak-proof secondary container and proceed to 1.5.

1.2. **Adherent cells seeded in cell culture plates and chamber slides:** Transport culture vessel to BSC (SOP, Use of the Class II Biological Safety Cabinet) and remove supernatant. Optional: wash cells with an appropriate washing buffer (e.g. PBS) to remove residual cell culture media. Place cell culture vessel in a leak-proof container and submerge in 10% formalin, 4% PFA, or 2% GTA. The leak-proof container must be completely filled with the fixative. For vessels with smaller wells (e.g. 96-well plates), add fixative to wells prior to submersion to prevent large air pockets. Rotate/agitate the container carefully to ensure the disinfectant comes into contact with all internal surfaces of the container. Proceed to 1.5.

1.3. **Cell pellets: <u>This method is only approved for the use of 10% formalin or 4% PFA.</u>** This inactivation protocol is used for cell suspensions. Transport cell culture vessel to BSC (SOP, Use of the Class II Biological Safety Cabinet) and transfer suspension cells into appropriate leak-proof tubes. Adherent cells must be detached by trypsinization or other methods to generate cell suspensions. Pellet cells by low-speed centrifugation. Optional: wash cell pellet with an appropriate washing buffer (e.g. PBS) and spin cells down after each washing step. Carefully pipette off residual liquid. Overlay cell pellet with 10% formalin or 4% PFA. Tube must be filled up to the top with fixative. Use a fresh, colored lid and apply fixative over the thread of the lid, doping the thread. The color of the lid indicates that the tube contains an inactivated sample. Proceed to 1.5.

1.4. **Viral particles:** This inactivation protocol is used for viral particles obtained from cell supernatants of infected cells and viral particles purified by ultracentrifugation. Supernatants from infected cells must be clarified by low-speed centrifugation to remove cell debris. Transport tubes containing viral particles to BSC (SOP, Use of the Class II Biological Safety Cabinet). Transfer desired volume of viral particles into an appropriate leak-proof tube and add the same volume of 20% formalin (e.g., 100 µL viral particles + 100 µL 20% formalin). The final formalin concentration must be 10%. The minimal total volume is 200 µL. Use a colored lid. The color of the lid indicates that the tube contains an inactivated sample. Apply fixative over the thread of the lid, doping the thread. Close the tube and vortex or shake it vigorously. **<u>All inner surfaces of the tube must be wetted with the 10% formalin solution.</u>** Proceed to 1.5.

1.5. Label plates, tubes, or containers with the following:

Your initials
Virus
Inactivating reagent
Start date and time of inactivation

1.6 Place the plates, tubes, or containers in the refrigerator for **<u>at least 6 hours</u>** to inactivate virus.

1.7. Complete electronic form *ALdehyde Inactivation Certificate* on Sharepoint (Inactivation Certificates).

1.8. **Removal of inactivated coverslips from the BSL-4 laboratory:** After the incubation time, transport cell culture plate containing the coverslips in the BSC. Transfer the coverslips into a leak-proof transport container completely filled with 10% formalin, 4% PFA, or 2% GTA. Remove transport container from

BSL-4 laboratory through the dunk tank following SOP, Removal of Non-viable Material from BSL-4 Containment Space.

1.9. **Removal of inactivated cell culture plates or chamber slides from the BSL-4 laboratory:** After the incubation time, remove leak-proof containers with inactivated plates and chamber slides submerged in the inactivating reagent from the BSL-4 laboratory through the dunk tank following SOP, Removal of Non-viable Material from BSL-4 Containment Space.

1.10. **Removal of inactivated cell pellets and viral particles from the BSL-4 laboratory:** After the incubation time, remove the leak-proof tubes containing the inactivated material in 10% formalin, 4% PFA, or 2% GTA from the BSL-4 laboratory through the dunk tank following SOP, Removal of Non-viable Material from BSL-4 Containment Space.

1.11. Collect any fixative used for inactivation within the BSL4 in a labeled hazardous waste container and place in the satellite accumulation area.

2. Forms

Aldehyde Inactivation Certificate (Inactivation Certificates).

3. Records Management

Correct performance of this SOP and removal of inactivated samples from BSL-4 containment must be documented by completing electronic form *Aldehyde Inactivation Certificate* on Sharepoint.

Supplementary Material S3

Procedure section of SOP *Inactivating BSL-4 material using heat*

This SOP does only contain the procedure section. It does not contain roles and responsibilities, required equipment and supplies, training requirements, safety requirements, personal protective equipment etc. The indicated links are deactivated.

1. Procedures and Instructions

The procedures described in this SOP are approved for filoviruses. Sodium dodecyl sulfate (SDS), lithium dodecyl sulfate (LDS), and guanidinium hydrochloride (GuHCl) must be maintained according to the manufacturer's specifications and must be within expiration date. An external thermometer placed in a tube of water in the heat block is used to confirm that the heat block is functioning properly.

All work with infectious material must be performed in a biological safety cabinet following SOP, Use of the Class II Biological Safety Cabinet. Removing inactivated samples will conform to SOP, Removal of Non-viable Material from BSL-4 Containment Space.

Approved inactivation methods

Type of Inactivation	Sample type	Maximum cell number/ maximum virus amount (filoviruses)	Maximum volume
Heat	Cells	up to $2.4x10^7$ cells	1 mL
	Supernatant, virus stock (FBS concentration must not exceed 10%)	up to $2.25x10^8$ $TCID_{50}$ units	1 mL

Table1. Approved inactivation parameters. All approved sample types are listed in the table. Any sample outside these parameters must be brought to the attention of EHS and cannot be removed from BSL-4 containment.

1.1. Switch on heat block and set temperature to 120°C/248°F. Place a tube filled with water in one of the wells and place external thermometer in this tube (test tube).
1.2. Prepare samples used for heat inactivation according to respective SOP.
1.3. In the BSC, transfer the cell lysates or virus solution (virus stock, supernatants) to be heat inactivated to fresh tubes. **These samples must contain a minimal final concentration of 1% SDS, 1% LDS, or 6 M GuHCl. The total volume of the samples must not exceed 1 mL.**
1.4. Close tubes using colored lids. Colored lids are used to discriminate between inactivated and live samples. Colored lids are exclusively used for inactivated samples. Vortex, invert, or shake tubes to make sure that all inner surfaces are wetted.
1.5. Wipe or spray tubes with an appropriate disinfectant before removing them from the BSC.
1.6. Place tubes containing samples in pre-heated heat block with the temperature set to 120°C/248°F. **Ensure the block temperature is at least 120°C (heat block reading) and the temperature in the test tube above 99°C (water boiling point is 100°C) as measured by the external thermometer before the sample tubes are placed into the heat block. Incubate samples for at least 10 min in the heat block.** If sample was previously frozen ensure sample is completely thawed before placing into the heat block.
1.7. Tubes must not be opened after heat inactivation in the BSL-4 lab.
1.8. Complete electronic form *Heat Inactivation Certificate* on SharePoint (Inactivation Certificates).
1.9. Remove samples from BSL-4 lab in accordance with SOP, Removal of Non-viable Material from BSL-4 Containment Space.

2. Forms

Heat Inactivation Certificate (Inactivation Certificates).

3. Records Management

Correct performance of this SOP and removal of heat-inactivated samples from BSL-4 containment must be documented by completing the electronic form *Heat Inactivation Certificate* on SharePoint.

Supplementary Material S4

November 17, 2021

Inactivation report: Inactivation of Nipah virus by TRIzol and TRIzol LS

Summary

The biosafety level 4 (BSL4) laboratory at Boston University's National Emerging Infectious Diseases Laboratories (NEIDL) facilitates the study of highly pathogenic viruses. Within the United States, BSL4 laboratories are regulated by the United States Centers for Disease Control and Prevention's (CDC) Division of Select Agents and Toxins (DSAT) and the United States Department of Agriculture (USDA). Complete inactivation of infectious BSL4 viruses, as governed by the Federal Select Agent Program (FSAP) regulations (7 CFR Part 331, 9 CFR Part 121.3, 42 CFR Part 73.3), is required before samples may be removed from a BSL4 laboratory for analysis at a lower containment level. This requires that the inactivated samples do not contain any replication competent virus. Inactivation can be achieved by chemical or physical procedures, many of which are well established, but these procedures must be appropriately validated at each facility to confirm a lack of infectivity/viability. FSAP regulations (September 2018) state that the validation of inactivation procedures only needs to be performed using a single member of any given family of viruses. Here we report verification and validation of inactivation of paramyxoviruses by TRIzol and TRIzol LS using Nipah virus (NiV), a member of the paramyxovirus family, as our exemplar. We used the NiV Bangladesh isolate for our studies because this virus can grow to high viral titers, as needed for this study.

TRIzol and TRIzol LS are two commonly used chemical mixes used for RNA extraction. These are routinely used to inactivate enveloped, negative sense RNA viruses, including BSL4 Select Agents such as NiV (Jensen et al., 2018; Kochel et al., 2017; Lo et al., 2010; Mire et al., 2016). Provided below are the methodology and results of testing we have performed in the NEIDL BSL4 to confirm the effectiveness of TRIzol and TRIzol LS at inactivating NiV.

As further detailed below, both TRIzol and TRIzol LS led to the complete inactivation of NiV in infected cells and supernatants, respectively, with a contact time of 10 minutes. The study was repeated one time with the same outcome.

Methodology

Use of columns to reduce toxicity of TRIzol and TRIzol LS

TRIzol and TRIzol LS are mixtures of phenol (CAS # 108-95-2), guanidine isothiocyanate (CAS # 593-84-0), and ammonium thiocyanate (CAS # 1762-95-4) in slightly different ratios. Due to the toxicity of these chemicals, samples containing TRIzol or TRIzol LS cannot be directly assayed for the presence of infectious virus. Size exclusion columns (Amicon Ultra-0.5 Centrifugal Filter Unit 10 kDa) were used to remove the components of TRIzol and TRIzol LS but maintain infectious virus. These Amicon columns

contain a cellulose-based filter that allows for the purification of products as small as 10 kilodaltons. For comparison, the individual NiV glycoproteins, F and G, are approximately 90 and 110 kilodaltons, respectively, and intact virus is much, much larger. Our previous analysis showed limited loss of Ebola virus due to purification over the Amicon columns, while eliminating the toxicity due to TRIzol and TRIzol LS (see report **Inactivation of Ebola virus by TRIzol and TRIzol LS**). Thus, these filters were predicted to efficiently also retain NiV, while facilitating the elimination of the much smaller components of TRIzol and TRIzol LS (the three components range in size from 0.08 to 0.12 kilodaltons). Our analysis showed limited virus loss due to purification over the Amicon columns (**Figure 1**), while eliminating the toxicity due to TRIzol and TRIzol LS (see **Figure 6**, mock + TRIzol, and **Figure 7**, mock + TRIzol LS).

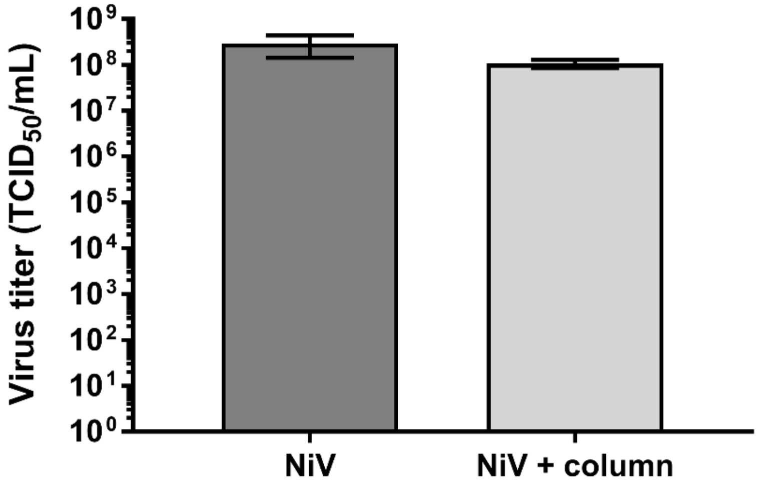

Figure 1. Analysis of virus loss due to Amicon column purification

Processing and analysis of samples

TRIzol (Invitrogen)

TRIzol is used to extract RNA from cells. To test the ability of TRIzol to inactivate NiV, $1x10^7$ Vero E6 cells seeded in T175 flasks were mock-infected or infected with NiV (isolate Bangladesh) at a multiplicity of infection (MOI) of 0.5 $TCID_{50}$ (50% Tissue Culture Infective Dose) units per cell (**Figure 2**, 1st infection). Two days post-infection, the cells were scraped into 20 mL of PBS, transferred into tubes, pelleted by low-speed centrifugation and resuspended in 1 mL DMEM or TRIzol. The samples were vortexed and incubated for 10 minutes at room temperature. Samples were purified using Amicon columns, per manufacturer's protocol. The samples were eluted in 0.5 mL of PBS.

To determine the infection rate, Vero E6 cells seeded in chamber slides were infected in parallel with NiV at the same MOI (MOI = 0.5). These infection control slides were fixed 2 days post-infection, at the time of virus inactivation, and analyzed for the presence of NiV by immunofluorescence analysis.

To determine the cell number of the T175 flasks at the time of inactivation, an extra flask with cells was incubated for the same time and used to count the cells (2.76×10^7 cells after 2 days).

The eluates form the Amicon columns were then used to infect 2×10^7 Vero E6 cells seeded in T175 flasks (**Figure 2**, 2nd infection). For the NiV-challenge sample, NiV was mixed with the column-purified eluate from TRIzol-treated non-infected cells and used to infect cells at an MOI of 0.01. The NiV-challenge sample was included to show that NiV can replicate in cells that were exposed to column-purified TRIzol cell lysates. The following samples were generated:

1. Mock cells (no virus; negative control)
2. Mock cells + TRIzol
3. Mock cells + TRIzol, NiV challenge (MOI of 0.01)
4. NiV infected cells
5. NiV infected cells + TRIzol

The cells from the second infection were incubated for 4 days and checked for signs of CPE (results are shown in **Figure 6**). At day 4 post-infection, cell supernatants were passaged onto fresh cells to further amplify the virus. To do this, cell supernatants were clarified by low-speed centrifugation and the entire supernatant was used to infect Vero E6 cells seeded in T175 flasks (**Figure 6**, 3rd infection). The next day, cell supernatants were removed and replaced with 35 mL of cell culture medium. The flasks were incubated for another 3 days and checked for CPE (**Figure 6**).

At day 4 post-infection, cell supernatants from the third infection (**Figure 2**, 3rd infection) were clarified by low-speed centrifugation and 0.2 mL was used to infect Vero E6 cells seeded in a 96-well plate (**Figure 2**, 4th infection). Two days post-infection, the cells were fixed with 10% formalin and stained for the presence of NiV using NiV-specific polyclonal antibodies (immunofluorescence analysis). See **Figure 2** for a pictorial representation of the methodology used to test NiV inactivation using TRIzol.

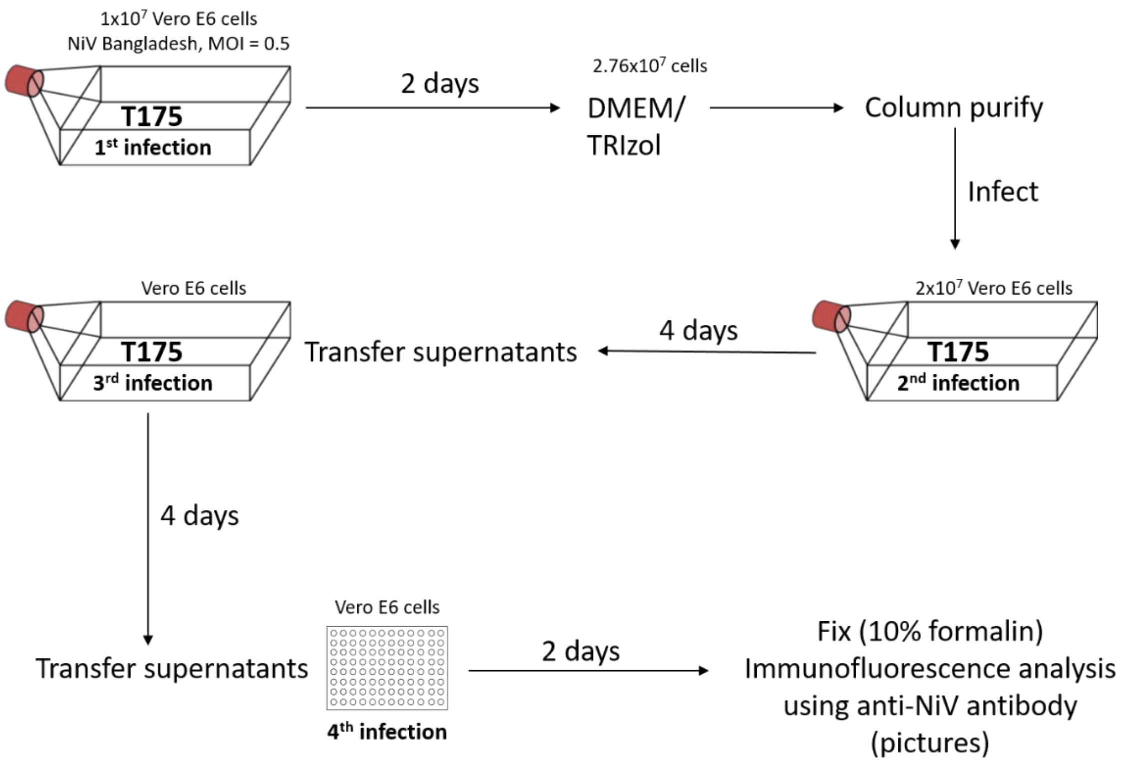

Figure 2. Experimental setup of testing inactivation of NiV with TRIzol.

TRIzol LS (Invitrogen)

TRIzol LS is used to extract RNA from liquid samples, including virus-containing cell supernatants. To test the ability of TRIzol LS to inactivate NiV, 250 µL of NiV stock (1.25×10^7 $TCID_{50}$ units) in DMEM + 2% fetal bovine serum (FBS) or 250 µL DMEM + 2% FBS (mock control) were mixed with 750 µL of TRIzol LS. The ratio of TRIzol LS to the sample was 3:1 (vol/vol), as recommended by the manufacturer. The 3:1 ratio was also shown to completely inactivate filoviruses in our previous filovirus inactivation study (see Appendix I SCI-SOP-0050). The samples were vortexed and incubated for 10 minutes at room temperature. TRIzol LS-containing samples were purified using Amicon columns, per manufacturer's protocol. The samples were eluted in 0.5 mL of PBS and used to infect 2×10^7 Vero E6 cells seeded in T175 flasks (**Figure 3**, 1st infection). The NiV-challenge sample (see below) was included to show that NiV can replicate in cells that were exposed to column-purified TRIzol LS. The positive and negative controls were the same as described above for TRIzol inactivation.

The following samples were generated:

6. DMEM + TRIzol LS
7. DMEM + TRIzol LS, NiV challenge (MOI of 0.01)
8. NiV stock + DMEM
9. NiV stock + TRIzol LS

The cells were incubated for 4 days and checked for signs of CPE (results are shown in **Figure 7**). At day 4 post-infection, cell supernatants were passaged onto fresh cells to further amplify the virus. To do this, cell supernatants were clarified by low-speed centrifugation and the entire supernatant was used to infect Vero E6 cells seeded in T175 flasks (**Figure 3**, 2nd infection) overnight. The next day, supernatant was removed and 35 mL of fresh media were added. The flasks were incubated for 3 additional days and checked for CPE.

At day 4 post-infection, cell supernatants were clarified by low-speed centrifugation and 0.2 mL were used to infect Vero E6 cells seeded in a 96-well plate (**Figure 3**, 3rd infection). Two days post-infection, the cells were fixed with 10% formalin and stained for the presence of NiV using NiV-specific polyclonal antibodies (immunofluorescence analysis). See **Figure 3** for a pictorial representation of the methodology used to test NiV inactivation using TRIzol LS.

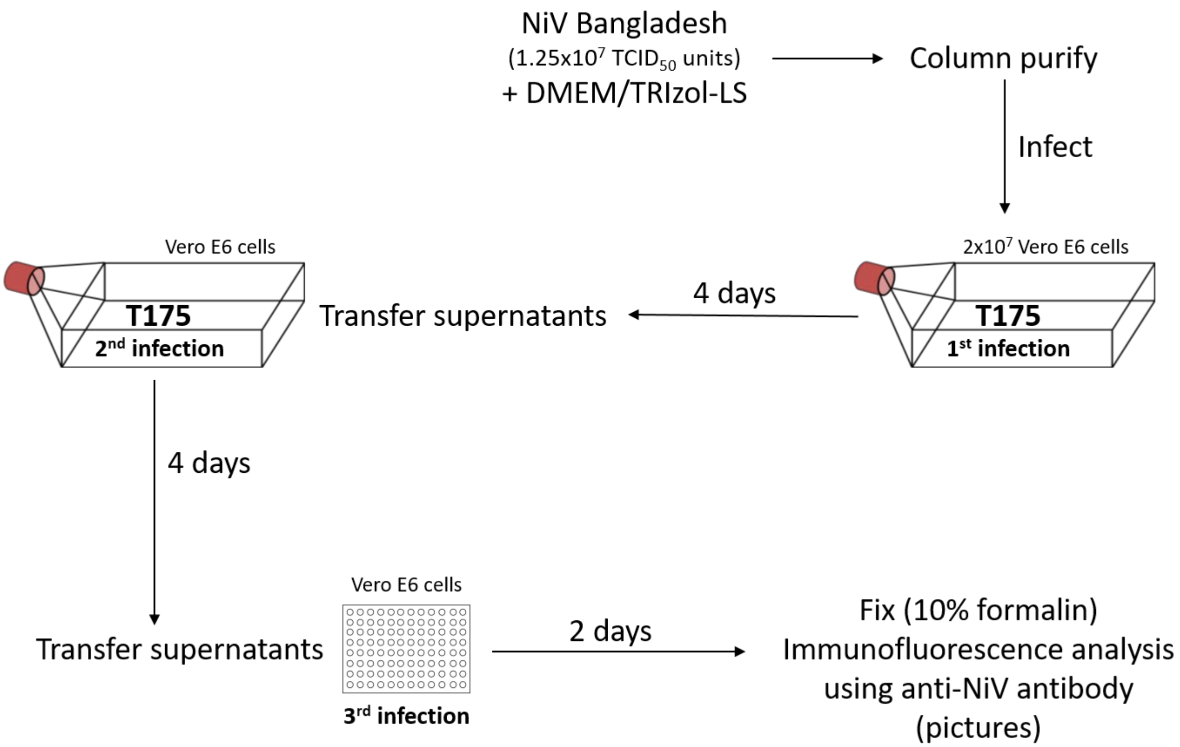

Figure 3. Experimental setup of testing inactivation of NiV with TRIzol LS

Readout

The two methods used to determine the presence of NiV in samples were cytopathic effect (CPE) and immunofluorescence analysis using polyclonal antibodies directed against NiV proteins. All flasks and plates were monitored for CPE, which is a hallmark of NiV infection. CPE is characterized by cell fusion and syncytia formation and ultimately cell death. Thus, cell monolayers exhibiting CPE include those

which have cell syncytia and loss of cells due to cell death. **Figure 4A** shows zoomed-in examples of samples with no CPE, a low-to-moderate amount of CPE (CPE patches circled in red) and lots of CPE.

Because immunostaining to visualize NiV-infected cells requires fixation which precludes further incubation and analysis of samples, this staining was only performed on samples used to determine the initial infection rate (described above) and the final infections in the 96-well plates (**Figure 2**, 4th infection and **Figure 3**, 3rd infection). Briefly, immunostaining was performed with cells fixed with 10% formalin. The used polyclonal antibodies recognize NiV proteins. We used a green fluorescent secondary antibody, meaning only cells that contain NiV proteins should fluoresce green following excitation of the fluorophores (see **Figure 4B** for zoomed-in example pictures).

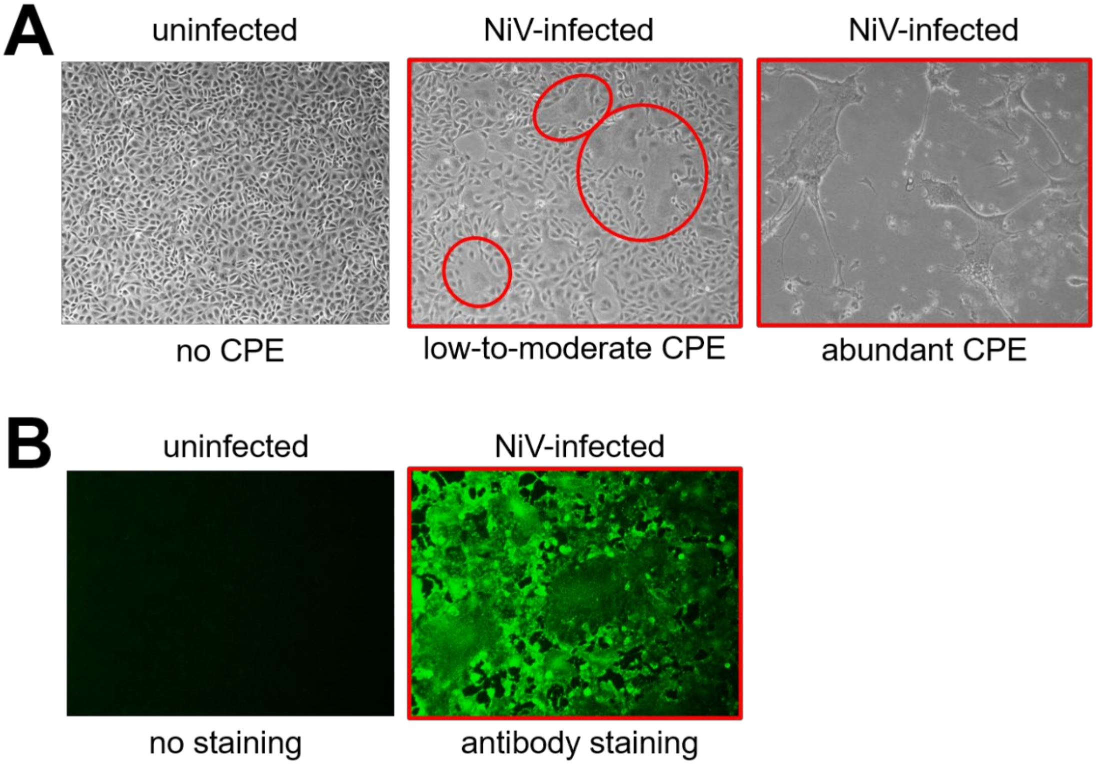

Figure 4. Examples of readouts for detecting NiV. (A) Brightfield microscopy to show the cytopathic effect (CPE) caused by NiV infection. (B) Immunofluorescence analysis using polyclonal antibodies against NiV.

Results

Initial infection rate

The initial NiV infection rates of cells for the TRIzol inactivation study were determined by infecting slides with the same MOI for 2 days (described above in Methodology). After 2 days, cells were fixed and

immunofluorescence was performed to detect the presence of NiV proteins. As shown in **Figure 5**, virtually all cells were infected with NiV (compare NiV staining to DAPI staining which stains the nuclei of all cells). Since the cells seeded in the T175 flasks were infected at the same MOI, these results indicate the infected cells used for the TRIzol inactivation studies were infected at a high infection rate.

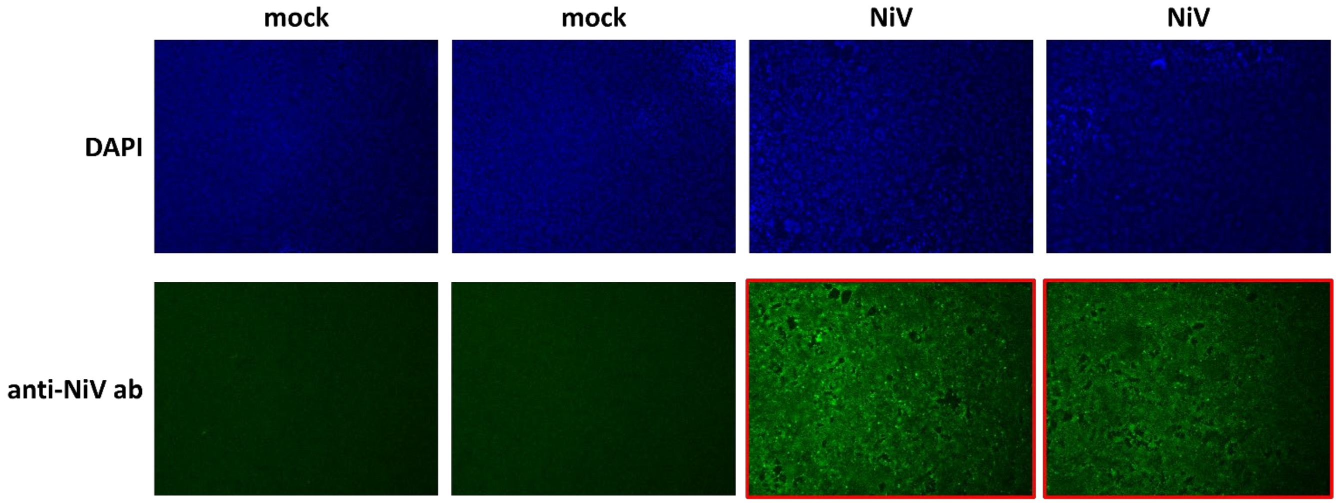

Figure 5. Initial infection rates at 2 days post-infection. Vero E6 cells seeded in chamber slides were left uninfected (mock) or infected with NiV isolate Bangladesh at a multiplicity of infection (MOI) of 0.5 $TCID_{50}$ units per cell. Two days post-infection, the cells were fixed with 10% formalin and subjected to immunofluorescence analysis using polyclonal antibodies directed against NiV. DAPI = blue staining of cellular nuclei. NiV = green staining of NiV proteins, indicative of infected cells. Red border = infected cells. ab, antibody.

Analysis of samples

Figures 6 and 7 show the brightfield images (for determination of CPE) at the indicated time points and the results of the immunofluorescence analysis to show the presence of NiV (green images in the right column). As expected, the mock sample (**sample 1**) showed no CPE or NiV staining. The positive control (**NiV infected cells, sample 4**) showed robust NiV infection, both by CPE and NiV-specific staining. The mock + TRIzol and mock + TRIzol LS samples (**samples 2 and 6**, respectively) showed no CPE, indicating that the Amicon columns worked well at eliminating these chemicals and their associated toxicity. Likewise, the NiV-challenge samples (**samples 3 and 7**) showed ample NiV infection, both by CPE and NiV staining, indicating that treatment of cells with samples containing TRIzol or TRIzol LS that had been column purified did not inhibit the ability of the cells to be subsequently infected with NiV (i.e. these cells were still capable of being infected). **Critically, samples 5 and 9 (NiV + TRIzol and NiV + TRIzol LS) did not show any CPE or NiV-specific staining, indicating that these samples did not contain any infectious/viable virus.**

Figures 6 and 7 show representative images of those taken during the course of the experiment (all pictures are representative of at least 2 pictures taken).

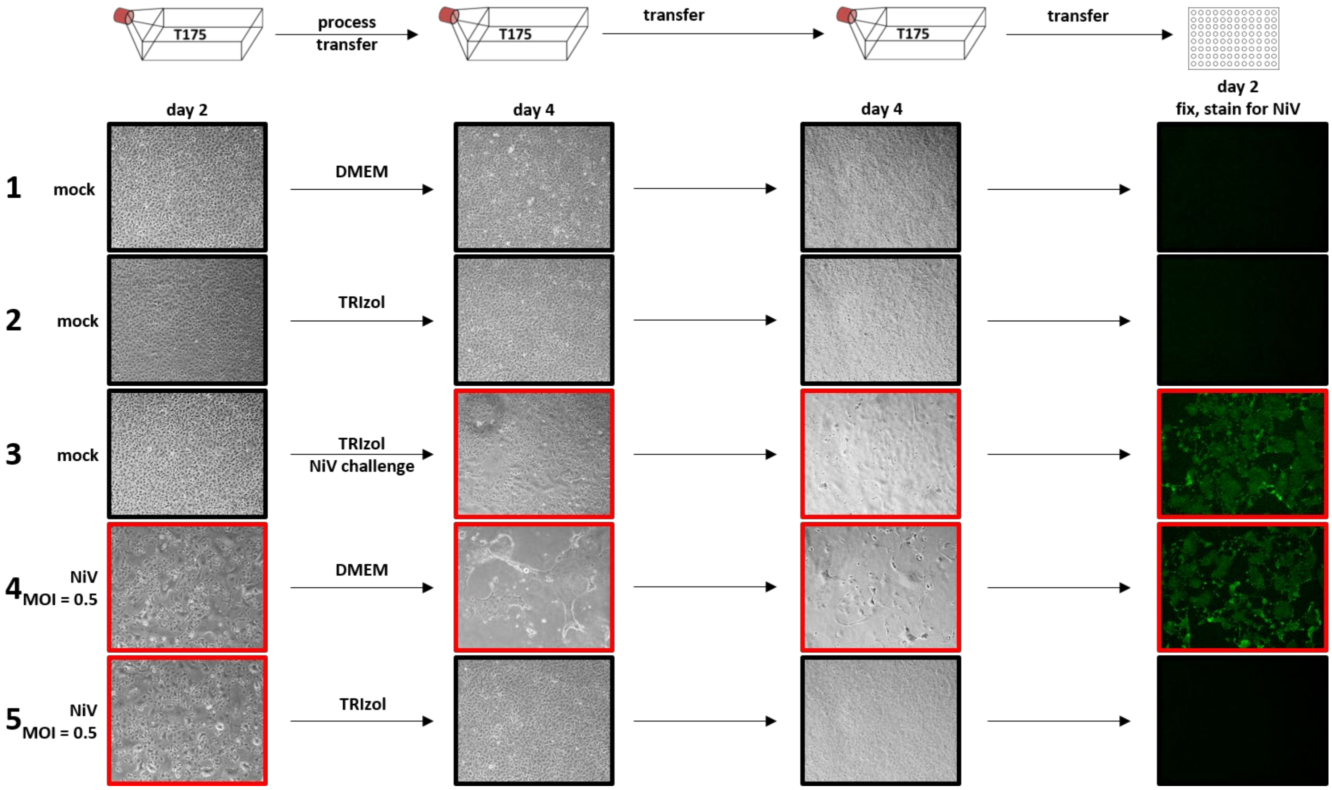

Figure 6. Inactivation of NiV with TRIzol. Black border = no CPE and negative immunofluorescence staining indicative of the lack of NiV infection. **Red border** = CPE and positive immunofluorescence indicative of NiV infection.

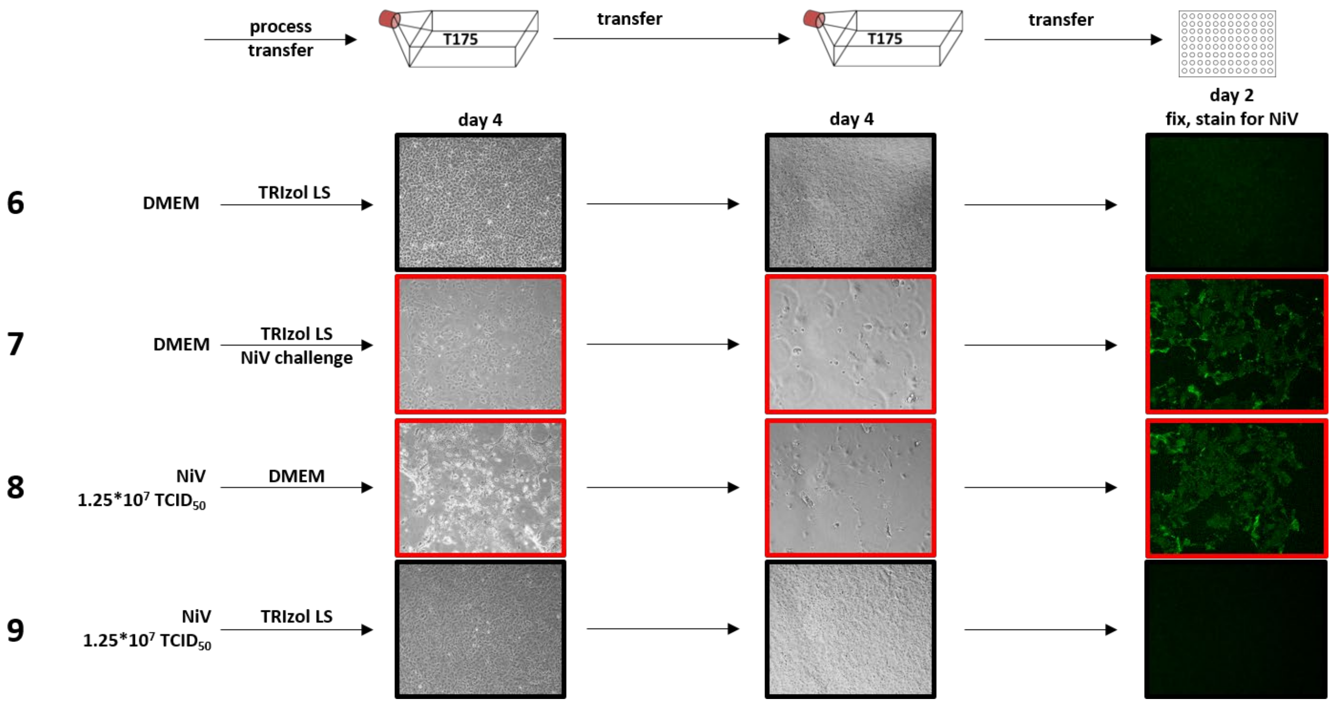

Figure 7. Inactivation of NiV with TRIzol LS. Black border = no CPE and negative immunofluorescence staining indicative of the lack of NiV infection. **Red border** = CPE and positive immunofluorescence indicative of NiV infection.

Conclusion

Our data show that 1 mL TRIzol is sufficient to completely inactivate 2.76x10^7 cells infected with NiV after a 10-minute incubation time at room temperature. We also showed that 1.25x10^7 $TCID_{50}$ units of NiV in a volume of 250 µL are completely inactivated after the addition of 750 µL TRIzol LS (3:1 ratio of TRIzol LS to sample volume) and a 10-minute incubation at room temperature. We were not able to detect live NiV after these treatments, even after serial passaging and long incubation times (a total of 10 days of incubation). There are not many published reports explicitly describing NiV inactivation as of the time of writing this report, but TRIzol and TRIzol LS treatment have previously been used to inactivate NiV (Jensen et al., 2018; Kochel et al., 2017; Lo et al., 2010; Mire et al., 2016). In addition, our data are in line with our previous report describing inactivation of Ebola virus using TRIzol and TRIzol LS and with published data on Ebola virus inactivation (Alfson and Griffiths, 2018; Blow et al., 2004; Haddock et al., 2016; Kochel et al., 2017).

Based on these inactivation data, we developed an agent-specific inactivation SOP that describes the critical inactivation steps and informs about the maximum virus loads tested. For documentation purposes, we also developed an inactivation certificate that will accompany the inactivated samples.

Limit of detection analysis

To determine the sensitivity of our testing procedure, a limit of detection analysis was performed. Briefly, small amounts of virus (1 and 10 $TCID_{50}$ units of NiV) were used to infect $2x10^7$ Vero E6 cells seeded in T175 flasks (**Figure 8**, 1st infection). The cells were incubated for 4 days, and the entire clarified supernatants were used to infect fresh Vero E6 cells seeded in T175 flasks overnight (**Figure 8**, 2nd infection). The next day, cell supernatants were removed and replaced with cell culture medium. 4 days post-infection, the supernatants were clarified, and 0.2 mL used to infect Vero E6 cells seeded in a 96-well plate for immunofluorescence analysis as described above (**Figure 8**, 3rd infection). The cells were fixed at day 2 post-infection. The results of this study are shown in **Figure 9**.

Due to the very low infection dose, there was moderate CPE during the 1st infection after 4 days (**Figure 9**). After passaging the supernatants onto fresh cells, some of the samples showed clear CPE after 4 days (**Figure 8**, red borders). These samples were also positive in the immunofluorescence analysis. In summary, we reliably observed virus infection when the cells were infected with a $TCID_{50}$ unit of 10 but not lower amounts of virus (**Figure 9**). These results show that our NiV detection assays are sufficient to detect extremely small amounts of virus (10 $TCID_{50}$ units) and are comparable to what we observed previously for Ebola virus.

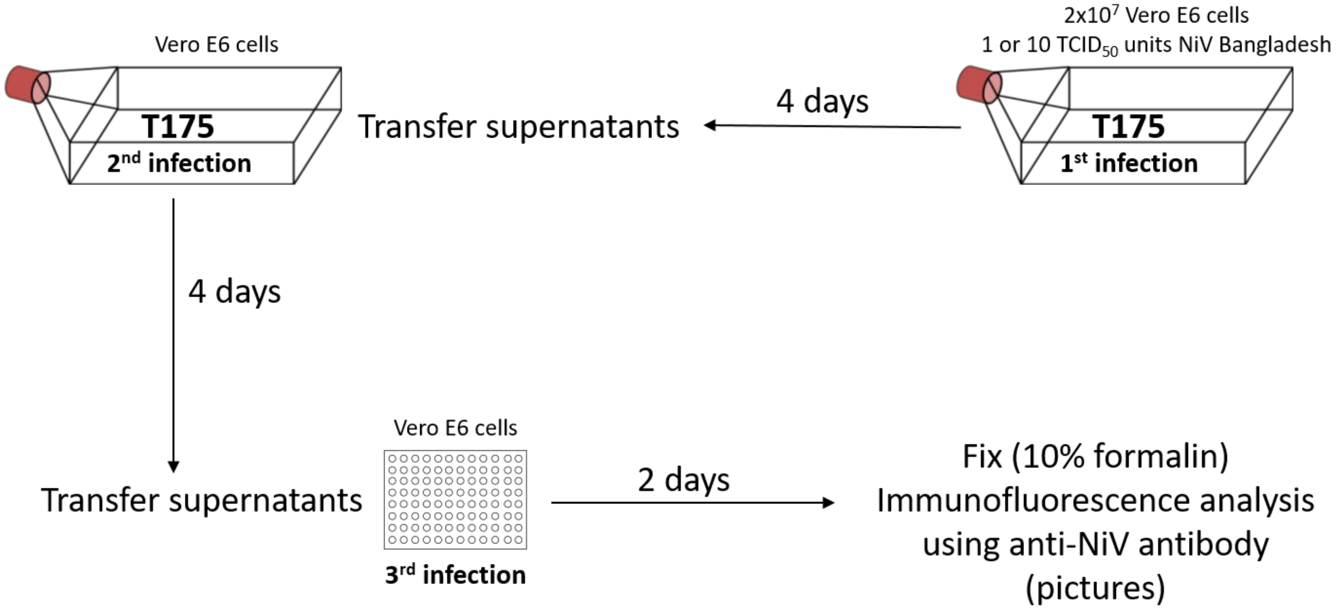

Figure 8. Experimental setup to determine the detection limit of infectious NiV.

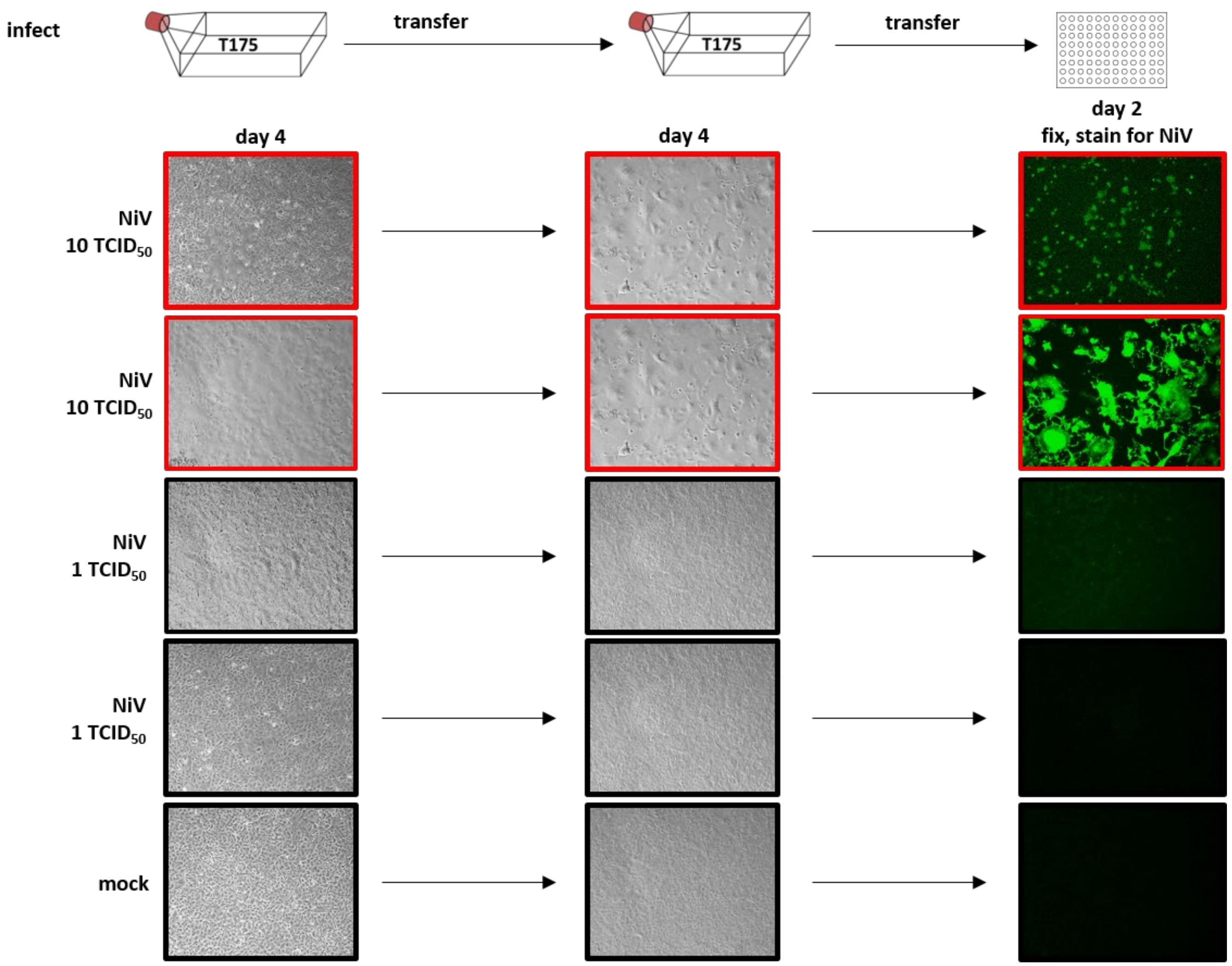

Figure 9. Limit of detection analysis. Black border = no CPE and/or negative immunofluorescence staining indicative of the lack of NiV infection. **Red border** = CPE and/or positive immunofluorescence indicative of NiV infection.

Additional documents

Appendix I – SOP, Inactivating BSL-4 material using TRIzol or TRIzol LS

Appendix II – TRIzol/TRIzol LS Inactivation Certificate

References

Alfson, K.J., Griffiths, A., 2018. Development and Testing of a Method for Validating Chemical Inactivation of Ebola Virus. Viruses 10.

Blow, J.A., Dohm, D.J., Negley, D.L., Mores, C.N., 2004. Virus inactivation by nucleic acid extraction reagents. J Virol Methods 119, 195-198.

Haddock, E., Feldmann, F., Feldmann, H., 2016. Effective Chemical Inactivation of Ebola Virus. Emerging infectious diseases 22.

Jensen, K.S., Adams, R., Bennett, R.S., Bernbaum, J., Jahrling, P.B., Holbrook, M.R., 2018. Development of a novel real-time polymerase chain reaction assay for the quantitative detection of Nipah virus replicative viral RNA. PLoS One 13, e0199534.

Kochel, T.J., Kocher, G.A., Ksiazek, T.G., Burans, J.P., 2017. Evaluation of TRIzol LS Inactivation of Viruses. Applied Biosafety 22, 52-55.

Lo, M.K., Miller, D., Aljofan, M., Mungall, B.A., Rollin, P.E., Bellini, W.J., Rota, P.A., 2010. Characterization of the antiviral and inflammatory responses against Nipah virus in endothelial cells and neurons. Virology 404, 78-88.

Mire, C.E., Satterfield, B.A., Geisbert, J.B., Agans, K.N., Borisevich, V., Yan, L., Chan, Y.P., Cross, R.W., Fenton, K.A., Broder, C.C., Geisbert, T.W., 2016. Pathogenic Differences between Nipah Virus Bangladesh and Malaysia Strains in Primates: Implications for Antibody Therapy. Sci Rep 6, 30916.

Certificate of Inactivation of BSL-4 materials using TRIzol or TRIzol LS

TRIzol/TRIzol LS Inactivation Certificate

This form is used in combination with SOP, Inactivating BSL-4 material using TRIzol or TRIzol LS for inactivation of BSL-4 material. Briefly, samples are incubated with TRIZol or TRIZol LS for 10 minutes, which is an effective, verified method of virus inactivation. Complete this form and save for records. (To ensure full compatibility, this form must be completed with Adobe Acrobat Reader or Acrobat Pro. The authors and publisher are not affiliated with Adobe.)

Person inactivating samples:	Date & time of Inactivation:	Comments:	Inactivation Certificate #:

Adherent monolayer or cell pellets:

☐ Sample does not exceed $2x10^7$ cells/mL of TRIzol

TRIzol lot number(s)	

Samples:

Quantity	Sample type	Volume TRIzol added	Virus

Cell lysates:

☐ Sample does not exceed $2x10^7$ cells lysed in at least 1 mL approved lysis buffer.

Supernatants

☐ Amount of Virus 1 before addition of TRIzol LS does not exceed $2.25x10^8$ TCID50 units per 250 µL.

☐ Amount of Virus 2 before addition of TRIzol LS does not exceed $1.25x10^7$ $TCID_{50}$ units per 250 µL.

☐ Amount of Virus 3 before addition of TRIzol LS does not exceed $9.9x10^7$ $TCID_{50}$ units per 250 µL.

TRIZol LS lot number(s)	

Samples:

Quantity	Sample volume	Volume TRIzol LS added	Virus

Samples were treated according to SOP "Inactivating BSL-4 material using TRIzol or TRIzol LS" - check as you go:

☐ TRIzol/TRIzol LS added to sample

☐ Incubated for at least 10 minutes for inactivation as measured by a lab timer

☐ Sample transferred to new tube

☐ TRIzol/TRIzol LS added to thread of colored lid, used colored lid to close tube

☐ Sample vortexed in new tube

Electronic signature of person performing inactivation

Electronic signature of PI or designee

The PI/designee signer verifies that the lab worker who performed the SOP has appropriate training and has fulfilled all required documentation steps to assure the SOP has been followed.

Principal Investigator

Date/Time added to Dunk Tank

- ☐ Number of samples removed from dunk tank matches above
- ☐ Verify inactivation by lid color or container label

Electronic signature of person adding samples to dunk tank

Date/Time removed from dunk tank

Signature (Person removing from dunk tank)
